# Visualizing and Quantifying Intracellular Behavior and Abundance of the Core Circadian Clock Protein PERIOD2

**DOI:** 10.1016/j.cub.2016.05.018

**Published:** 2016-07-25

**Authors:** Nicola J. Smyllie, Violetta Pilorz, James Boyd, Qing-Jun Meng, Ben Saer, Johanna E. Chesham, Elizabeth S. Maywood, Toke P. Krogager, David G. Spiller, Raymond Boot-Handford, Michael R.H. White, Michael H. Hastings, Andrew S.I. Loudon

**Affiliations:** 1 Neurobiology Division, Medical Research Council (MRC) Laboratory of Molecular Biology (LMB), Francis Crick Avenue, Cambridge CB2 0QH, UK; 2 Faculty of Life Sciences, University of Manchester, Oxford Road, Manchester M13 9PT, UK

## Abstract

Transcriptional-translational feedback loops (TTFLs) are a conserved molecular motif of circadian clocks. The principal clock in mammals is the suprachiasmatic nucleus (SCN) of the hypothalamus. In SCN neurons, auto-regulatory feedback on core clock genes *Period* (*Per*) and *Cryptochrome* (*Cry*) following nuclear entry of their protein products is the basis of circadian oscillation [1, 2]. In *Drosophila* clock neurons, the movement of dPer into the nucleus is subject to a circadian gate that generates a delay in the TTFL, and this delay is thought to be critical for oscillation [3, 4]. Analysis of the *Drosophila* clock has strongly influenced models of the mammalian clock, and such models typically infer complex spatiotemporal, intracellular behaviors of mammalian clock proteins. There are, however, no direct measures of the intracellular behavior of endogenous circadian proteins to support this: dynamic analyses have been limited and often have no circadian dimension [5, 6, 7]. We therefore generated a knockin mouse expressing a fluorescent fusion of native PER2 protein (PER2::VENUS) for live imaging. PER2::VENUS recapitulates the circadian functions of wild-type PER2 and, importantly, the behavior of PER2::VENUS runs counter to the *Drosophila* model: it does not exhibit circadian gating of nuclear entry. Using fluorescent imaging of PER2::VENUS, we acquired the first measures of mobility, molecular concentration, and localization of an endogenous circadian protein in individual mammalian cells, and we showed how the mobility and nuclear translocation of PER2 are regulated by casein kinase. These results provide new qualitative and quantitative insights into the cellular mechanism of the mammalian circadian clock.

## Highlights

• Reporter mouse is used for real-time fluorescent imaging of mammalian clock protein PER2
• In contrast to *Drosophila*, localization of PER2 is not subject to circadian gating
• Circadian abundance, mobility, and intracellular dynamics of native PER2 are quantified
• Casein kinase1 controls nucleocytoplasmic mobility of PER2 alongside circadian period

## Graphical Abstract

**Figure:**
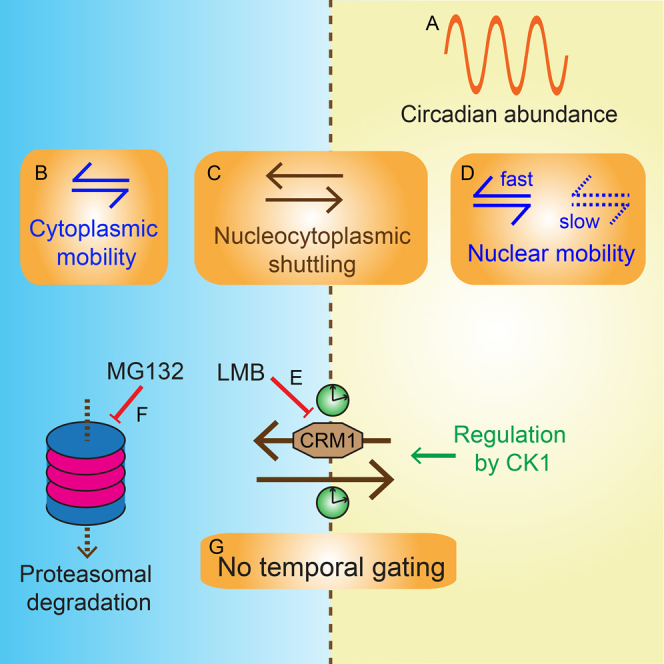

## Teaser

Smyllie et al. use a fluorescent reporter mouse to image the circadian dynamics of PER2, a key component of the circadian clock in the suprachiasmatic nucleus and fibroblasts. They reveal marked divergence of the mechanisms of mouse and fly clock cells, and they provide quantitative data to support reappraisal of current models of the mammalian clock.

## Results

### Generation and Validation of PERIOD2::VENUS Mouse

**Figure 1:**
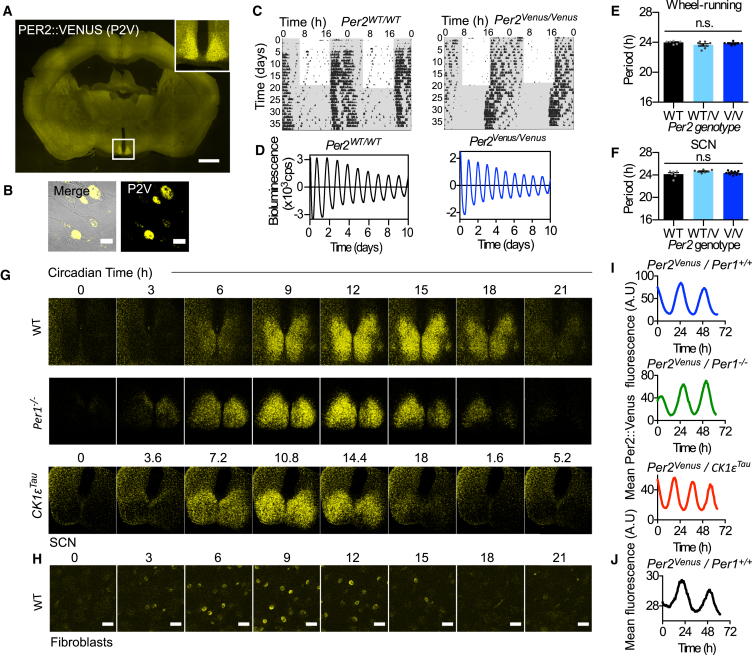
PER2::VENUS Fusion Protein Is a Competent Circadian Clock Protein Suitable for Real-Time Imaging (A) PER2::VENUS fluorescence across the mouse brain, at the peak time of SCN expression (ZT12). Inset shows a close up of the SCN. Scale bar, 1 mm. (B) Bright-field and fluorescence confocal images show *Per2*^*Venus*^ lung fibroblasts. Scale bar, 20 μm. (C) Representative, double-plotted wheel-running actograms for *Per2*^*WT*^ (left) and *Per2*^*Venus*^ (right) animals. Mice were entrained on a 12:12 LD cycle, followed by a schedule of constant conditions (dim red light, represented by shaded gray). (D) Representative, de-trended *Per1*-*luc* bioluminescence rhythms of SCN slices from *Per2*^*WT*^ (left) and *Per2*^*Venus*^ (right) mice are shown. (E) Mean ± SEM circadian periods for wheel-running are shown (n_WT_ = 6; n_WT/V_ = 8; n_V/V_ = 7). (F) Mean ± SEM circadian periods for SCN slices (n_WT_ = 6; n_WT/V_ = 7; n_V/V_ = 9). One-way ANOVA revealed no significant effect for either measure. (G) Snapshots from confocal real-time imaging show PER2::VENUS fluorescence in representative *Per2*^*Venus*^ (top panel), Per1 null (middle panel), and *CK1ε*^*Tau*^ (lower panel) in SCN slices. (H) Snapshots from confocal real-time imaging show PER2::VENUS in fibroblasts. Scale bar, 20 μm. (I) Mean fluorescence measures from recordings in (G) are shown. (J) Mean fluorescence measures from recordings in (H) are shown. See also Figure S1, Table S1, and Movie S1.

We used homologous recombination to knock in a fluorescent tag at the *Per2* locus, an equivalent strategy to that used for the PER2::LUC mouse, which is known to exhibit wild-type (WT) PER2 behavior [8]. Venus was fused to exons 19–23 of *mPer2* (Figure S1A). The presence of PER2::VENUS protein expression was confirmed by fluorescence microscopy in the brain and in lung fibroblasts (Figures 1A and 1B). As well as strong fluorescence in the suprachiasmatic nucleus (SCN), limited expression was observed in the piriform cortex, thalamus, and hippocampus (Figure S1B). Importantly, the spatial distribution of PER2::VENUS co-localized completely with PER2 immunoreactivity (-ir) in *Per2*^*WT*/*Venus*^ SCN (Figures S1C–S1E).

To test for normal circadian function in *Per2*^*Venus*/*Venus*^ animals, we first assessed wheel-running behavior. They entrained effectively to a 12-hr light/12-hr dark schedule (12:12 LD), and they exhibited consolidated circadian activity patterns of wheel-running when placed in constant conditions (Figures 1C, S1F, and S1G). There were no significant differences between WT and *Per2*^*Venus*^ mice in the distribution, structure, or robustness (measured by non-parametric circadian rhythm analysis) of circadian behavior. After crossing with *Per1*-*luc* reporter mice, *Per2*^*Venus*/*Venus*^ SCN organotypic slices expressed robust, high-amplitude circadian bioluminescence rhythms (Figures 1D and S1H). The circadian periods of behavioral and SCN rhythms were not significantly different between WT and *Per2*^*Venus*/*Venus*^ mice (Figures 1E and 1F). Thus, PER2::VENUS did not compromise molecular pacemaking in the SCN or effective circadian control over behavior. To confirm that *Per2*^*Venus*^ did not encode a loss-of-function mutation, *Per2*^*Venus*^ mice were crossed to *Per1*^−/−^ mice. In the absence of PER1, WT PER2 is a necessary and sufficient component of the circadian pacemaker [9]. *Per2*^*Venus*/*Venus*^, *Per1*^−/−^ mice exhibited robust and sustained wheel-running and SCN bioluminescence rhythms (Figures S1I and S1J), with comparable periods to *Per2*^*WT*/*WT*^, *Per1*^−/−^ mice (Figure S1K). Thus, *Per2*^*Venus*^ encodes a functional allele of PER2. *Per2*^*Venus*^ mice were then crossed with *CK1ε*^*Tau*^ mutants to test whether PER2::VENUS can interact with CK1ε, a key modulator of PER2 stability and circadian period [10]. In WT animals, the *CK1ε*^*Tau*/*Tau*^ mutation shortened period from ∼24 to ∼20 hr (Figures S1I, S1J, and S1L) [10]. *Per2*^*Venus*/*Venus*^ mice showed comparable acceleration of SCN and behavioral rhythms. Thus, *Per2*^*Venus*^ encodes an endogenous fusion protein that functions competently within the mammalian clock.

### Intracellular Circadian Dynamics of Endogenous PERIOD2

We next analyzed the rhythmicity of the PER2::VENUS protein. A clear circadian oscillation of PER2::VENUS abundance was detected by western blot in temperature-entrained lung fibroblasts (Figures S1M and S1N). PER2::VENUS was also highly and rhythmically expressed in the SCN (Figures S1O and S1P). At the peak of PER2 expression (zeitgeber time 12 [ZT12]), in the SCN, PER2::VENUS was detected in effectively all arginine vasopressin (AVP)-immunoreactive (ir) and vasoactive intestinal peptide (VIP)-ir neurons but in <10% of gastrin-releasing peptide (GRP)-ir neurons (Figure S2; Table S1). At the trough of the cycle (CT0), only a few AVP-ir cells expressed PER2::VENUS (Table S1). We next tested the utility of PER2::VENUS as a real-time circadian reporter, using confocal microscopy. Both SCN slices and lung fibroblasts exhibited stable, high-amplitude circadian oscillations of fluorescence throughout 70 hr of recording (Figures 1G and 1H; Movie S1). In the SCN, PER2::VENUS peaked appropriately at 1 and 4 hr, respectively, after *Cry1*-*luc* and *Per1*-*luc* (Figures S3A and S3B). Circadian expression of PER2::VENUS was well defined in the SCN with a *Per1*^−/−^ background and accurately reported period shortening by the *CK1ε*^*Tau*^ mutation. Thus, PER2::VENUS is a high-fidelity real-time reporter of the behavior of an endogenous clock protein in SCN neurons and fibroblasts.

We next determined the macrodynamics of PER2::VENUS. Using cycloheximide to inhibit translation in SCN slices, we revealed that PER2::VENUS has a half-life of∼2 hr, comparable to that of PER2::LUC (Figures S3C–S3E) [10]. Consistent with proteasomal degradation of Per2^WT^ [11], application of the proteasomal inhibitor MG132 at CT12 increased PER2::VENUS levels above those of vehicle-treated lung fibroblasts (Figure S3F) and SCN slices (Figures S3G and S3H). The nuclear export inhibitor leptomycin B, applied at CT12, significantly increased the half-life of PER2::VENUS, suggesting that nuclear export facilitates degradation (i.e., PER2 is subject to degradation in the cytoplasm; Figures S3I and S3J). Moreover, synchronization between cells in SCN slices was reduced following nuclear export blockade, suggesting that timely nuclear export and degradation are necessary for transcriptional-translational feedback loop (TTFL) timing.

**Figure 2:**
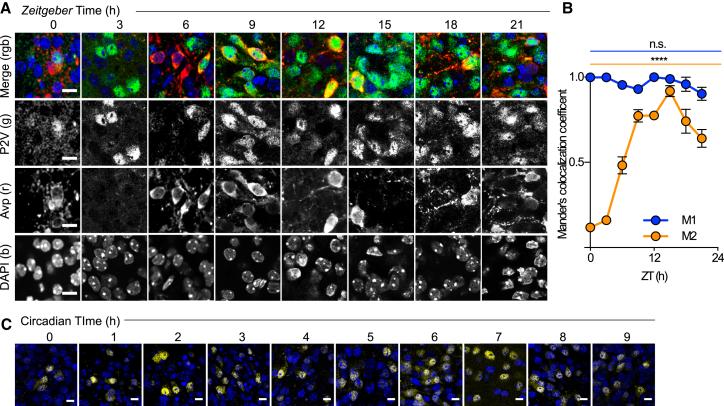
Circadian Subcellular Localization of PER2::VENUS (A) Representative confocal images of SCN neurons from brain sections taken from *Per2*^*Venus*^ animals across the LD cycle. PER2::VENUS -positive (green) localization was compared with cytoplasmic immunostaining for AVP (red) and nuclear staining of DAPI (blue). Scale bar, 20 μm. (B) Co-localization of PER2::VENUS and DAPI assessed by Mander’s coefficient analysis (M1, blue, co-localization of PER2::VENUS with DAPI; M2, yellow, co-localization of DAPI with PER2::VENUS). Note that M2 changes across the day because the overall level of PER2::VENUS changes; thus, the proportion of DAPI nuclei containing PER2::VENUS changes. (C) *Per2*^*Venus*^ SCN slices were fixed, counterstained with DAPI (blue), and imaged at different time points during the rising phase (CT0–CT9) of the PER2 circadian cycle. Each image shows representative neurons found at that time point, rather than a representative field of view at that time point. See also Figure S2.

Gated nuclear entry of Per protein is considered a pivotal feature of circadian timekeeping. We examined the subcellular localization of PER2::VENUS in the SCN at different points across the LD cycle. Although the overall abundance of PER2::VENUS changed across the cycle, its subcellular localization did not (Figure 2A). Mander’s M1 coefficient indicated almost complete co-localization of Venus with nuclear DAPI signal at all phases (Figure 2B): at no point was PER2::VENUS excluded from the nucleus, with exclusively cytoplasmic localization. To discount the possibility of a transient gate for cytoplasmic retention and nuclear entry, cellular localization was monitored in living SCN slices imaged during the rising phase (CT0–CT9) of PER2 expression. Again, when detected, PER2::VENUS was observed in the nucleus at all time points (Figure 2C). This was also the case in fibroblasts, where (weak) cytoplasmic fluorescence oscillated in phase with strong nuclear fluorescence (Figures S3K–S3M). Thus, in marked contrast to the temporal gating that delays *Drosophila* Per entry to the nucleus to late subjective day (4, 5), the mouse ortholog, PER2, is not subject to compartmental circadian gating in SCN neurons or fibroblasts and nuclear accumulation occurs progressively.

### Quantitative Analysis of PERIOD2 Intracellular Mobility and Abundance

**Figure 3:**
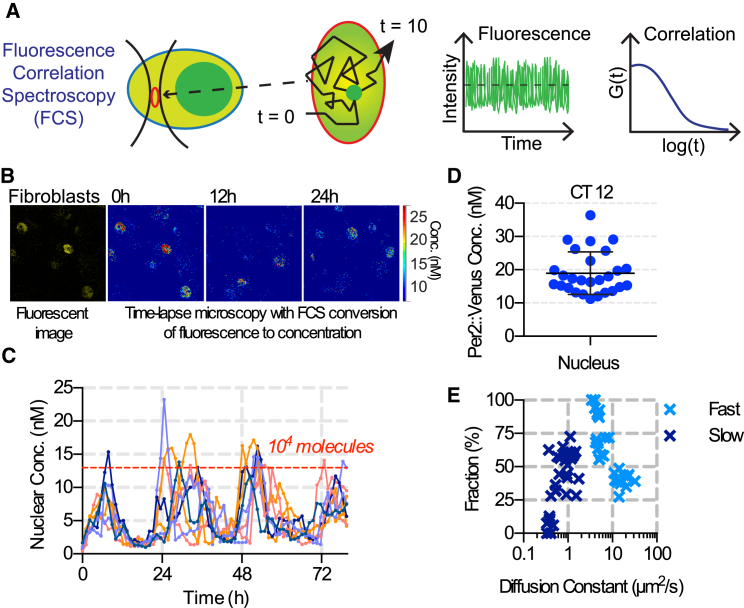
FCS of PER2::VENUS Protein in Fibroblasts (A) Schematic diagram illustrating the FCS procedure. PER2::VENUS fluorescence was monitored within a small confocal volume. Individual fluorescent molecules passed through the volume at a given rate. The fluorescence signal of all of the molecules in the volume was followed through time. The concentration and rate of movement of molecules were calculated by auto-correlating the fluorescence signal. (B) Fluorescence images and FCS-calibrated quantification of PER2::VENUS concentration in skin fibroblasts are shown. (C) Circadian variation of nuclear concentration of PER2::VENUS over time for eight representative cells. Images were collected every 6 min but every tenth image was analyzed; thus, there is a data point every 1.12 hr. (D) PER2::VENUS concentration in the nucleus at CT12 (4–6 hr after temperature synchronization) is shown (mean ± SEM). (E) FCS data fit to a two-component diffusion model, color coded by component (fast, light blue; slow, dark blue), are shown. See also Figures S3 and S4.

Fluorescence correlation spectroscopy (FCS) was used to measure mobility and molecular abundance of PER2::VENUS in skin fibroblasts (Figure 3A). Circadian changes in PER2::VENUS concentration were observed in fibroblast nuclei (Figure 3B); but, importantly, calibration of fluorescence intensity to FCS-calculated concentration per nuclear volume enabled absolute quantification of the molecular abundance of PER2::VENUS across the circadian cycle (Figures 3C and S4A–S4D). This revealed a 10-fold amplitude cycle, with peak expression of ∼15,000 molecules per nucleus (>90% of cellular total; Figure 3D). Interestingly, when FCS-derived auto-correlations were fit to a two-component diffusion model, a bimodal distribution of diffusion coefficients was determined for nuclear PER2. This indicates that it exists in at least two molecular/dynamic states, possibly as bound and unbound fractions (Figure 3E). The more mobile fraction had a diffusion coefficient of ∼8 μm^2^s^−1^. Furthermore, at CT12 the number of nuclear PER2::VENUS molecules, their diffusion rates, and the proportion displaying the slowly diffusing component were not significantly different in *CK1ε*^*Tau*^ fibroblasts compared to WT (Figures S3E–S3G). This suggests that PER2 mobility within the nucleus is not affected in the mutant. Thus, we have presented the first quantitative measures of intracellular dynamics of a mammalian circadian clock protein.

**Figure 4:**
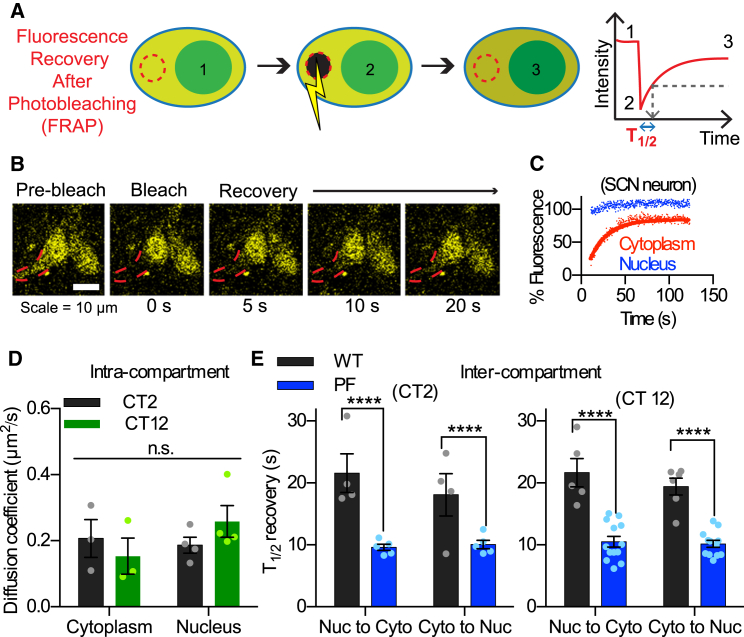
FRAP Reveals Role of CK1 in Regulating PER2 Mobility in SCN Neurons (A) A schematic diagram illustrating FRAP as follows: (1) select region and measure pre-bleach fluorescence, (2) photobleach region, and (3) monitor the recovery of fluorescence. The T_1/2_ is calculated from the recovery curve. (B) Snapshots from a FRAP experiment show cytoplasmic photobleaching and fluorescence recovery in neurons in an SCN slice. (C) Fluorescence recovery for a bleached cytoplasmic region (red) and unbleached nuclear region (blue). The latter shows no change in fluorescence over the time course. (D) FRAP-derived diffusion coefficients (mean ± SEM) within SCN nucleus and cytoplasm were comparable at both peak and trough (CT2 and CT12) of the cycle (n_CT2,cyto_ = 3 slices, n_CT12,cyto_ = 4, n_CT2,nuc_ = 3, and n_CT12,nuc_ = 4; two-way ANOVA). Diffusion coefficients were estimated from FRAP-derived T_1/2_ measures. (E) FRAP-derived T_1/2_ (mean ± SEM) calculated from total cytoplasm (Nuc to Cyto) and total nucleus (Cyto to Nuc) FRAP of SCN neurons at CT2 (left) and CT12 (right). Treatment with PF670462 (1 μM) significantly decreased T_1/2_ times for PER2::VENUS (n_CT2,WT,cyto_ = 4 slices, n_CT2,WT,nuc_ = 4, n_CT2,PF,cyto_ = 5, n_CT2,PF,nuc_ = 5, n_CT12,WT,cyto_ = 5, n_CT12,WT,nuc_ = 6, n_CT12,PF,cyto_ = 13, and n_CT12,PF,nuc_ = 12; two-way ANOVA with Tukey’s comparison, ^∗∗∗∗^p < 0.0001). See also Figure S4 and Movie S2.

Fluorescence recovery after photobleaching (FRAP) was used to further examine the intracellular behavior of PER2::VENUS. Data obtained by photobleaching of fibroblast nuclei agreed with the FCS-based calculations that intranuclear PER2 mobility was unaffected by the *CK1ε*^*Tau*^ mutation (Figure S4H). Furthermore, after bleaching nuclear fluorescence, full recovery of nuclear fluorescence did not occur within the experimental time frame, confirming that the bulk (>90%) of PER2::VENUS was nuclear (Figure S4I). FRAP also was measured in SCN slices (Figure 4), yielding diffusion coefficients within cytoplasm or nucleus of ∼0.2 μm^2^s^−1^. This is comparable to the slow diffusing component revealed by FCS in fibroblasts (Figure 3E). The diffusion coefficient was calculated for both cytoplasm and nucleus, at CT12 and CT2. Intracompartment mobility was comparable for both cytoplasm and nucleus at both time points (Figure 4D). PER2 mobility was not reduced in the nucleus, which may have been expected for a transcriptional regulator. Translocation of PER2 into the nucleus is critical for the circadian TTFL [6, 7]. Thus, T_1/2_ of FRAP was measured after photobleaching either the whole nucleus or cytoplasm (Figures 4B and 4C; Movie S2) to quantify between-compartment mobility. Importantly, this showed that there is no compartmental or temporal restriction over the mobility of PER2::VENUS (Figure 4E), in agreement with the confocal time-lapse imaging data (i.e., movement between compartments was comparable in both directions).

CK1δ/ε activity is an important regulator of the TTFL, where CK1-mediated phosphorylation of PER proteins licenses them for ubiquitination and proteasomal degradation. To test whether CK1 modulates PER2 mobility, we treated SCN slices with CK1 inhibitor PF670462, which slowed *Per1*-*luc* oscillations to 28.6 ± 0.30 hr (n = 10, p < 0.01 versus pre-treatment). Intracompartment mobility was unaffected by treatment with PF670462 (Figures S4J and S4K); but, surprisingly, both at CT2 and CT12 it significantly decreased the T_1/2_ of FRAP (Figure 4E; i.e., the rates of PER2::VENUS bi-directional translocation were accelerated). Furthermore, the magnitude of the increases in T_1/2_ were consistent across time points (CT2/CT12) and direction of movement (Figure S4K). Thus, decreasing CK1 activity increased PER2::VENUS translocation rates in an unbiased manner. In conclusion, low CK1ε/δ activity, which significantly lengthened the period of the SCN TTFL, was associated with accelerated nucleo-cytoplasmic shuttling of PER2::VENUS.

## Discussion

The PER2::VENUS mouse enables direct qualitative and quantitative observations of the complex spatiotemporal behavior of the PER2 protein, in a physiologically relevant setting. Our results suggest a pivotal role for the dynamic balance of nuclear entry and export for the determination of circadian period.

The PER2::VENUS allele was validated as clock competent, a property shared with PER2::LUC [8, 12]. Luciferase reporter systems, however, are not suitable for measuring fast events, such as nucleo-cytoplasmic shuttling of proteins. They produce a dim signal necessitating a long integration time for detection, and their inherent requirement for luciferin substrate can generate luciferase-chemistry-dependent artifacts. Fluorescent proteins do not suffer from these problems, but there are, of course, some potential limitations, including altered stability of the endogenous protein. The half-life of PER2::VENUS was similar to PER2::LUC, suggesting that the Venus tag did not alter the stability of the PER2 protein. PER2::LUC had a slightly shorter half-life, but this is in line with published literature reporting that luciferase-tagged reporters have a shorter half-life than their endogenous counterparts [13]. The PER2::VENUS half-life may be a more accurate estimate of PER2 stability, as it is a direct measure of the protein, rather than using enzymatic luciferase activity, which is an indirect measure. Prolonged fluorescence imaging can cause both phototoxicity and photobleaching, but for PER2::VENUS we found that it was possible to image SCN slices and fibroblast cultures over at least 70 hr without loss of fluorescence or circadian competence. Although there was limited detection of cytoplasmic PER2::VENUS in fibroblasts, photobleaching of the entire cytoplasm only reduced the nuclear fluorescence by <2% at CT12; thus, it is unlikely to contribute to the overall behavior of PER2 in this cell type. Overall, PER2::VENUS is a useful and faithful reporter for monitoring PER2 dynamics over shorter and longer timescales.

In contrast to the *Drosophila* ortholog, dPer, which accumulates in the cytoplasm prior to nuclear entry [3, 4], PER2 is not subject to a circadian gate in SCN neurons and in fibroblasts. This contrasting behavior may be explained by the proteins having different hetero-dimerization partners (dTim and CRY, respectively). Our data do not preclude nuclear gating of other clock factors, but there is, so far, no evidence for this. For example, snapshot immunostaining of PER1 in SCN highlights nuclear expression and no cytoplasmic restriction [14]. Progression of the TTFL in the mammalian clock, therefore, is not dependent on gated nuclear translocation; rather, it is achieved by graded nuclear accumulation of the negative regulators.

PER2::VENUS enabled us to quantify the number of PER2 molecules per cell and determine the dynamic changes in protein mobility. At CT12, PER2::VENUS was present at a concentration of ∼15–20 nM in fibroblast nuclei, equating to >10,000 molecules. Interestingly, stochastic simulations of the mammalian circadian clock predicted that stability within the virtual TTFL required >4,000 molecules [15]. Thus, our real observations are of the same order of magnitude and can inform future development of in silico models. The *Per2*^*Venus*^ mouse also facilitates analysis of how the intracellular behavior of PER2 directs the properties of the clock. Both FRAP and FCS revealed diffusion coefficients principally in the range of 0.1 to 1.0 μm^2^s^−1^. This is broadly compatible with data from other dynamic transcription factor proteins [16]. FCS also identified a more mobile fraction in fibroblasts, with a coefficient of ∼10 μm^2^s^−1^. The obtained fast and slow diffusion constants (∼10 and ∼1 μm^2^s^−1^), as well as free and bound fractions, are comparable to estimates made by FCS for the in vivo binding of glucocorticoid receptor [17].

Numerous studies link CK1 to the regulation of PER2 function and localization [18, 19, 20]. Both *CK1ε*^*Tau*^ and *hPer2*^*S662G*^ are mutants that have a short circadian period, with altered phosphorylation of PER2. The former exhibits rapid clearance of PER2 from the nucleus [10, 21] and the latter decreased nuclear retention [22, 23, 24]. These are two aspects of the same phenomenon. Our data demonstrate that CK1 contributes to the translocation of PER2 through the nuclear pores. Thus, CK1 is a critical regulator of PER2 mobility and circadian period, although the contribution of various types of mutation to short periods is not fully resolved [25, 26].

In conclusion, PER2::VENUS operates as a functional circadian protein, and it is sufficient to sustain timekeeping in the absence of WT Per proteins. We believe that the fundamental observations we have presented will support a significant reappraisal of the mammalian clock mechanism and provide valuable observational data on an endogenous clock protein that will inform the development of enhanced quantitative models of cellular circadian timekeeping.

## Author Contributions

N.J.S. and V.P. contributed equally to this work. N.J.S. designed, performed, and analyzed all SCN-related experiments. V.P. contributed to the generation and initial behavioral validation of the mouse, with input from E.S.M., Q.-J.M., and R.B.-H. J.B. conducted the FCS experiments with input from M.R.H.W. and D.G.S. B.S. managed breeding and generated primary skin fibroblasts for FCS experiments. J.E.C. supervised breeding and conducted wheel-running experiments. T.P.K. performed western blotting experiments with PER2::VENUS fibroblasts. N.J.S., M.H.H., V.P., and A.S.I.L. wrote the manuscript with input from Q.-J.M., J.B., D.G.S., and E.S.M.

## Supplemental Information

Document S1. Figures S1–S4, Table S1, and Supplemental Experimental Procedures

Movie S1. PER2::VENUS Rhythms in SCN Slices and Skin Fibroblasts, Related to Figure 1 Representative time-lapse recordings of PER2::VENUS fluorescence, recorded by confocal microscopy. Scale bar, 100 μm.

Movie S2. FRAP after Cytoplasmic or Nuclear Photobleaching, Related to Figure 4 Left: cytoplasmic photbleaching. Right: nuclear photobleaching. Representative time-lapse recordings represent 60 s of fluorescence recovery. Location of bleach is indicated by white outline.

Document S2. Article plus Supplemental Information

## References

[1] Shearman L.P., Sriram S., Weaver D.R., Maywood E.S., Chaves I., Zheng B., Kume K., Lee C.C., van der Horst G.T.J., Hastings M.H., Reppert S.M. (2000). Interacting molecular loops in the mammalian circadian clock. Science.

[2] Koike N., Yoo S.H., Huang H.C., Kumar V., Lee C., Kim T.K., Takahashi J.S. (2012). Transcriptional architecture and chromatin landscape of the core circadian clock in mammals. Science.

[3] Meyer P., Saez L., Young M.W. (2006). PER-TIM interactions in living *Drosophila* cells: an interval timer for the circadian clock. Science.

[4] Shafer O.T., Rosbash M., Truman J.W. (2002). Sequential nuclear accumulation of the clock proteins period and timeless in the pacemaker neurons of *Drosophila* melanogaster. J. Neurosci..

[5] Tamanini F., Yagita K., Okamura H., van der Horst G.T.J. (2005). Nucleocytoplasmic shuttling of clock proteins. Methods Enzymol..

[6] Yagita K., Tamanini F., Yasuda M., Hoeijmakers J.H.J., van der Horst G.T.J., Okamura H. (2002). Nucleocytoplasmic shuttling and mCRY-dependent inhibition of ubiquitylation of the mPER2 clock protein. EMBO J..

[7] Yagita K., Yamaguchi S., Tamanini F., van Der Horst G.T.J., Hoeijmakers J.H.J., Yasui A., Loros J.J., Dunlap J.C., Okamura H. (2000). Dimerization and nuclear entry of mPER proteins in mammalian cells. Genes Dev..

[8] Yoo S.H., Yamazaki S., Lowrey P.L., Shimomura K., Ko C.H., Buhr E.D., Siepka S.M., Hong H.K., Oh W.J., Yoo O.J. (2004). PERIOD2:LUCIFERASE real-time reporting of circadian dynamics reveals persistent circadian oscillations in mouse peripheral tissues. Proc. Natl. Acad. Sci. USA.

[9] Maywood E.S., Chesham J.E., Smyllie N.J., Hastings M.H. (2014). The Tau mutation of casein kinase 1ε sets the period of the mammalian pacemaker via regulation of Period1 or Period2 clock proteins. J. Biol. Rhythms.

[10] Meng Q.J., Logunova L., Maywood E.S., Gallego M., Lebiecki J., Brown T.M., Sládek M., Semikhodskii A.S., Glossop N.R.J., Piggins H.D. (2008). Setting clock speed in mammals: the CK1 epsilon tau mutation in mice accelerates circadian pacemakers by selectively destabilizing PERIOD proteins. Neuron.

[11] Eide E.J., Woolf M.F., Kang H., Woolf P., Hurst W., Camacho F., Vielhaber E.L., Giovanni A., Virshup D.M. (2005). Control of mammalian circadian rhythm by CKIepsilon-regulated proteasome-mediated PER2 degradation. Mol. Cell. Biol..

[12] Tahara Y., Kuroda H., Saito K., Nakajima Y., Kubo Y., Ohnishi N., Seo Y., Otsuka M., Fuse Y., Ohura Y. (2012). In vivo monitoring of peripheral circadian clocks in the mouse. Curr. Biol..

[13] Millar A.J., Short S.R., Chua N.H., Kay S.A. (1992). A novel circadian phenotype based on firefly luciferase expression in transgenic plants. Plant Cell.

[14] Field M.D., Maywood E.S., O’Brien J.A., Weaver D.R., Reppert S.M., Hastings M.H. (2000). Analysis of clock proteins in mouse SCN demonstrates phylogenetic divergence of the circadian clockwork and resetting mechanisms. Neuron.

[15] Forger D.B., Peskin C.S. (2005). Stochastic simulation of the mammalian circadian clock. Proc. Natl. Acad. Sci. USA.

[16] Bagnall J., Boddington C., Boyd J., Brignall R., Rowe W., Jones N.A., Schmidt L., Spiller D.G., White M.R., Paszek P. (2015). Quantitative dynamic imaging of immune cell signalling using lentiviral gene transfer. Integr. Biol. (Camb.).

[17] Stasevich T.J., Mueller F., Michelman-Ribeiro A., Rosales T., Knutson J.R., McNally J.G. (2010). Cross-validating FRAP and FCS to quantify the impact of photobleaching on in vivo binding estimates. Biophys. J..

[18] Akashi M., Tsuchiya Y., Yoshino T., Nishida E. (2002). Control of intracellular dynamics of mammalian period proteins by casein kinase I epsilon (CKIepsilon) and CKIdelta in cultured cells. Mol. Cell. Biol..

[19] Takano A., Isojima Y., Nagai K. (2004). Identification of mPer1 phosphorylation sites responsible for the nuclear entry. J. Biol. Chem..

[20] Vielhaber E., Eide E., Rivers A., Gao Z.H., Virshup D.M. (2000). Nuclear entry of the circadian regulator mPER1 is controlled by mammalian casein kinase I epsilon. Mol. Cell. Biol..

[21] Dey J., Carr A.J.F., Cagampang F.R.A., Semikhodskii A.S., Loudon A.S.I., Hastings M.H., Maywood E.S. (2005). The tau mutation in the Syrian hamster differentially reprograms the circadian clock in the SCN and peripheral tissues. J. Biol. Rhythms.

[22] Toh K.L., Jones C.R., He Y., Eide E.J., Hinz W.A., Virshup D.M., Ptácek L.J., Fu Y.H. (2001). An hPer2 phosphorylation site mutation in familial advanced sleep phase syndrome. Science.

[23] Vanselow K., Vanselow J.T., Westermark P.O., Reischl S., Maier B., Korte T., Herrmann A., Herzel H., Schlosser A., Kramer A. (2006). Differential effects of PER2 phosphorylation: molecular basis for the human familial advanced sleep phase syndrome (FASPS). Genes Dev..

[24] Xu Y., Toh K.L., Jones C.R., Shin J.Y., Fu Y.H., Ptácek L.J. (2007). Modeling of a human circadian mutation yields insights into clock regulation by PER2. Cell.

[25] Vanselow K., Kramer A. (2007). Role of phosphorylation in the mammalian circadian clock. Cold Spring Harb. Symp. Quant. Biol..

[26] Zhou M., Kim J.K., Eng G.W.L., Forger D.B., Virshup D.M. (2015). A Period2 phosphoswitch regulates and temperature compensates circadian period. Mol. Cell.

