# Supplementary material for: Visualizing and Quantifying Intracellular Behavior and Abundance of the Core Circadian Clock Protein PERIOD2
Source: Curr Biol. 2016 Jul 25;26(14):1880–6. doi: 10.1016/j.cub.2016.05.018 (PMC4963210; doi:10.1016/j.cub.2016.05.018)
Supplement: Document S1. Figures S1–S4, Table S1, and Supplemental Experimental Procedures [file mmc1.pdf]

Current Biology, Volume *26*

Supplemental Information

Visualizing and Quantifying Intracellular Behavior and Abundance of the Core Circadian Clock Protein PERIOD2

Nicola J. Smyllie, Violetta Pilorz, James Boyd, Qing-Jun Meng, Ben Saer, Johanna E. Chesham, Elizabeth S. Maywood, Toke P. Krogager, David G. Spiller, Raymond Boot-Handford, Michael R.H. White, Michael H. Hastings, and Andrew S.I. Loudon

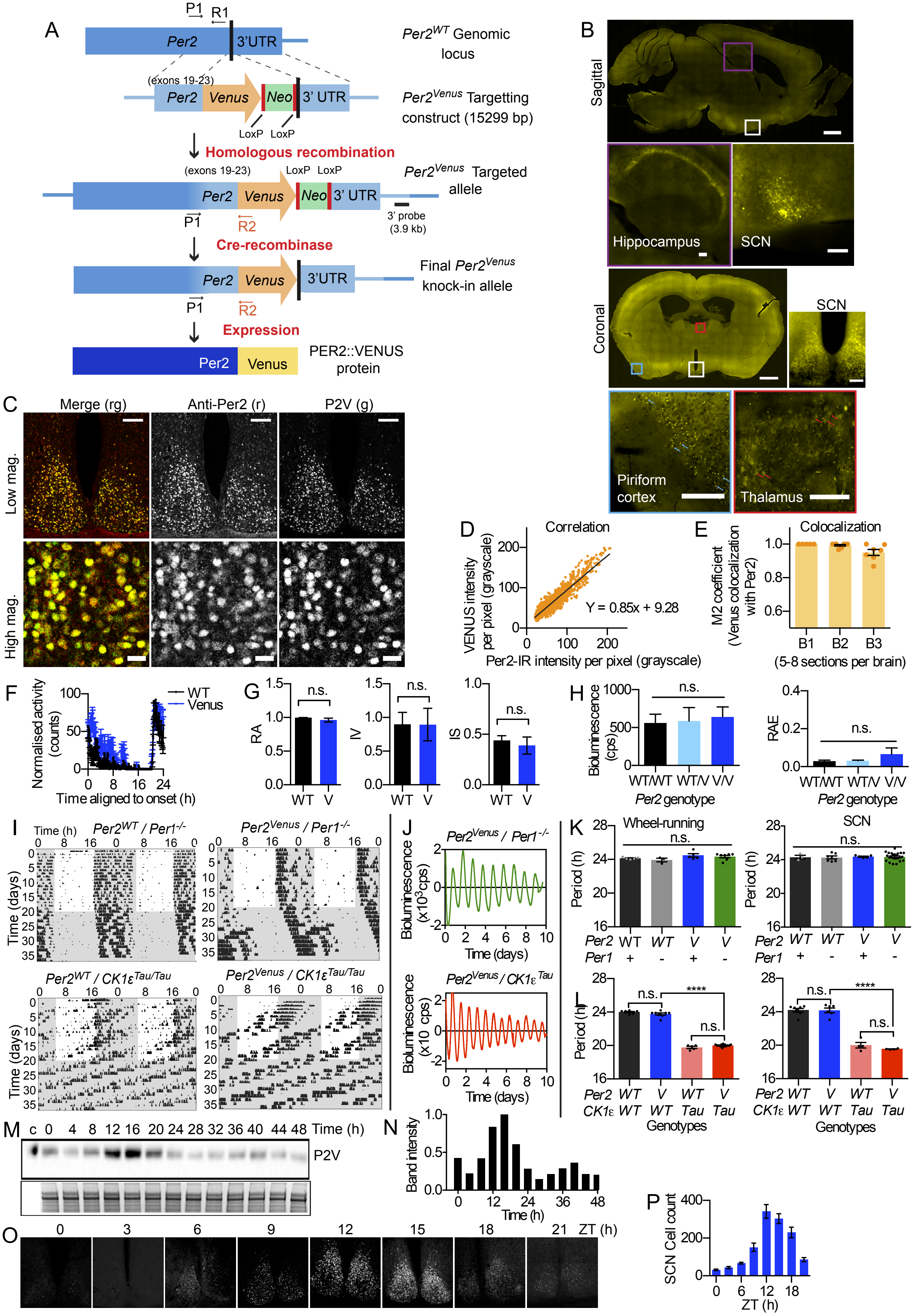
A
Per2 3'UTR
Per2WT Genomic locus
Per2 Venus Neo 3' UTR
Per2Venus Targetting construct (15299 bp)
Homologous recombination
Per2Venus Targeted allele
3' probe (3.9 kb)
Cre-recombinase
Final Per2Venus knock-in allele
Expression
PER2::VENUS protein
B
Sagittal
Hippocampus
SCN
Coronal
Piriform cortex
Thalamus
C
Merge (rg)
Anti-Per2 (r)
P2V (g)
Low mag.
High mag.
D
Correlation
Y = 0.85x + 9.28
VENUS intensity per pixel (grayscale)
Per2-IR intensity per pixel (grayscale)
E
Colocalization
M2 coefficient (Venus colocalization with Per2)
(5-8 sections per brain)
F
WT
Venus
Normalised activity (counts)
Time aligned to onset (h)
G
n.s.
RA
IV
IS
H
Bioluminescence (cps)
RAE
Per2 genotype
I
Per2WT / Per1-/-
Per2Venus / Per1-/-
Per2WT / CK1ε Tau/Tau
Per2Venus / CK1ε Tau/Tau
Time (days)
J
Bioluminescence
Time (days)
K
Wheel-running
SCN
Period (h)
L
****
Genotypes
M
Time (h)
P2V
N
Band intensity
Time (h)
O
ZT (h)
P
SCN Cell count
ZT (h)

Fig. S1. *Per2*^{Venus} mice express a functional fusion protein that is sufficient to drive circadian rhythms in gene expression and behavior. Related to Figure 1.

(A) A schematic diagram depicting the generation of the PER2::VENUS protein. The targetting construct contains exons 19-23 of *Per2* followed by the sequence for Venus, with floxed *neomycin* as a selection marker. The targeting construct was inserted into the *Per2* locus in the mouse by homologous recombination, where the 3' end of the endogenous gene was replaced with the targeting construct. *Neomycin* was later removed using Cre-recombinase. The final *Per2*^{Venus} knock-in allele resulted in the expression of the PER2::VENUS (P2V) fusion protein. Locations of genotyping primers are marked. P1+R1 gave a positive reaction in *Per2*^{WT} animals, whereas P1 and R2 gave a positive reaction in *Per2*^{Venus} animals. (B) Representative epifluorescence images showing PER2::VENUS expresson across the brain in both sagittal and coronal views. Snapshots of regions containing signficant PER2::VENUS expression are shown adjacent to the overview images. Their locations are marked with colored boxes. Fluorescence levels were particularly low in the piriform cortex and thalamus, so arrows highlighting cells have been included on the figure. (C) Representative confocal images showing the complete co-localisation of PER2::VENUS (green) with Per2-immunoreactivity (red) in SCN from *Per2*$^{WT/Venus}$, thus animals expressing both native *Per2*^{WT} and the *Per2*^{Venus} alleles. Upper panel shows the entire SCN (scale bar =100 μm) and lower panel shows close-up of individual cells (scale bar =20 μm). PER2::VENUS fluorescence intensity was highly correlated to Per2 immunostaining. (D) A representative correlation plot of pixel intensity in the red (Per2-IR) and green (PER2::VENUS) channels. (E) Mander's colocalization analysis indicated almost complete co-localization between the two channels (n =3). (F) Normalized, mean ± SEM daily DD wheel-running activity profile of WT ($n_{WT/WT}$ =5) and *Per2*$^{Venus/Venus}$ ($n_{V/V}$ =5) animals.

(G) Non-parametric Circadian Rhythm Analysis (NPCRA) of 12 days of DD wheel-running data. Relative Amplitude (RA; left), Intradaily Variability (IV; centre) and Interdaily Stability (IS, right) were not significantly different between WT and *Per2*^{Venus} (V) animals ($n_{WT/WT}$ =5; $n_{V/V}$ =5; unpaired T-test), thus circadian robustness of wheel-running activity was not affected by the presence of the *Per2*^{Venus} allele. (H) Circadian parameters of ampitude (left) and Relative Amplitude Error (RAE) (right) of SCN slices were not significantly affected by the *Per2*^{Venus} allele ($n_{WT/WT}$ =5; $n_{WT/V}$ =8; $n_{V/V}$ =9; one-way ANOVA). (I) Representative double-plotted actograms of wheel-running activity for *Per1*$^{-/-}$ (upper) and *CK1ε*$^{Tau/Tau}$ (lower) animals . Shaded areas mark times when lights are off. (J) Representative, de-trended Per1-luc bioluminescence rhythms from SCN slices. (K) In the absence of *Per1* expression (*Per1*$^{-/-}$; P1KO), SCN circadian period showed no significant effect of carrying the *Per2*^{Venus} allele (Behavior: $n_{WT,WT}$ =5; $n_{V,WT}$ =7; $n_{WT,P1KO}$ =3; $n_{V,P1KO}$ =7; SCN: $n_{WT,WT}$ =3; $n_{V,WT}$ =5; $n_{WT,P1KO}$ =7; $n_{V,P1KO}$ =24; two-way ANOVA). (L) Both wheel-running behavior (left) and SCN slices (right) from Per2$^{Venus/Venus}$ animals reported the short-period mutant phenotype of the *CK1ε*$^{Tau/Tau}$ (Tau) allele (Behavior: $n_{WT,WT}$ =6; $n_{V,WT}$ =9; $n_{WT,Tau}$ =5; $n_{V,Tau}$ =13; SCN: $n_{WT,WT}$ =7; $n_{V,WT}$ =6; $n_{WT,Tau}$ =4; $n_{V,Tau}$ =4; two-way ANOVA with Tukey's comparisons: **** $p < 0.0001$). All bar charts show mean ± SEM of group data. (M) Western blot showing rhythms of PER2::VENUS abundance in temperature-entrained *Per2*$^{Venus/Venus}$ fibroblasts. (N) Band intensity measures for western blot shown in (M). (O) Representative images of fluorescence in SCN sections across the day. (P) Mean ± SEM counts of PER2::VENUS-positive cells from SCN sections taken from mice at different time points across the light-dark cycle.

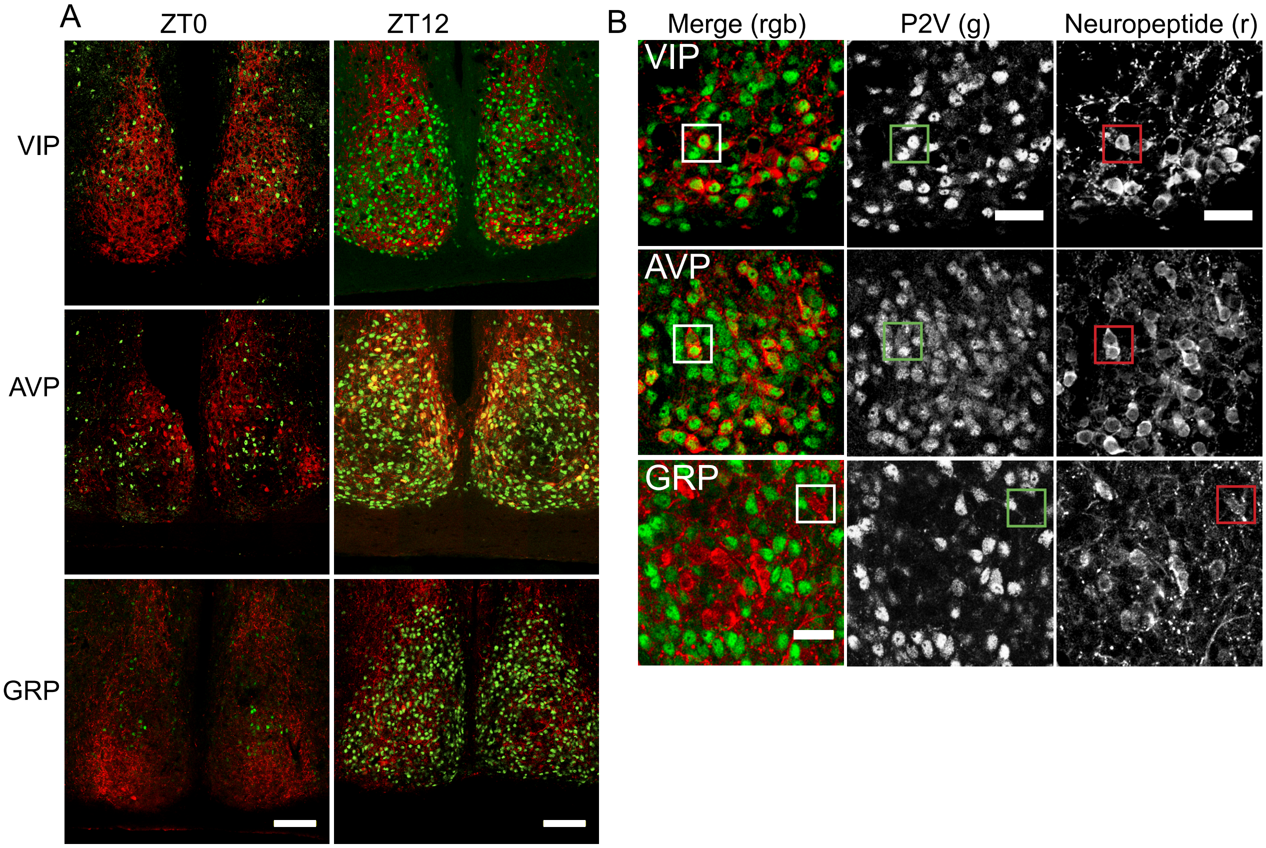

Fig. S2. Neurochemical identities of PER2::VENUS positive cells. Related to Figure 2. Representative images of SCN brain sections, showing immunofluorescence of SCN neuropeptides (red) vasoactive intestinal peptide (VIP), arginine vasopressin (AVP) and gastrin releasing peptide (GRP), colocalized with PER2::VENUS fluorescence (green) and counterstained with DAPI (blue). (A) Low-power images showing the whole SCN sections taken at ZT0 (left) and ZT12 (right). Scale bar = 100 uM. (B) Images showing "close-up" view of neuropeptide colocalization in the SCN at ZT12. The boxes (white for merged, red for neuropeptide and bue for DAPI channels), draw attention to positive colocalization of PER2::VENUS with VIP and AVP, but absence with GRP. Scale bar = 50 uM.

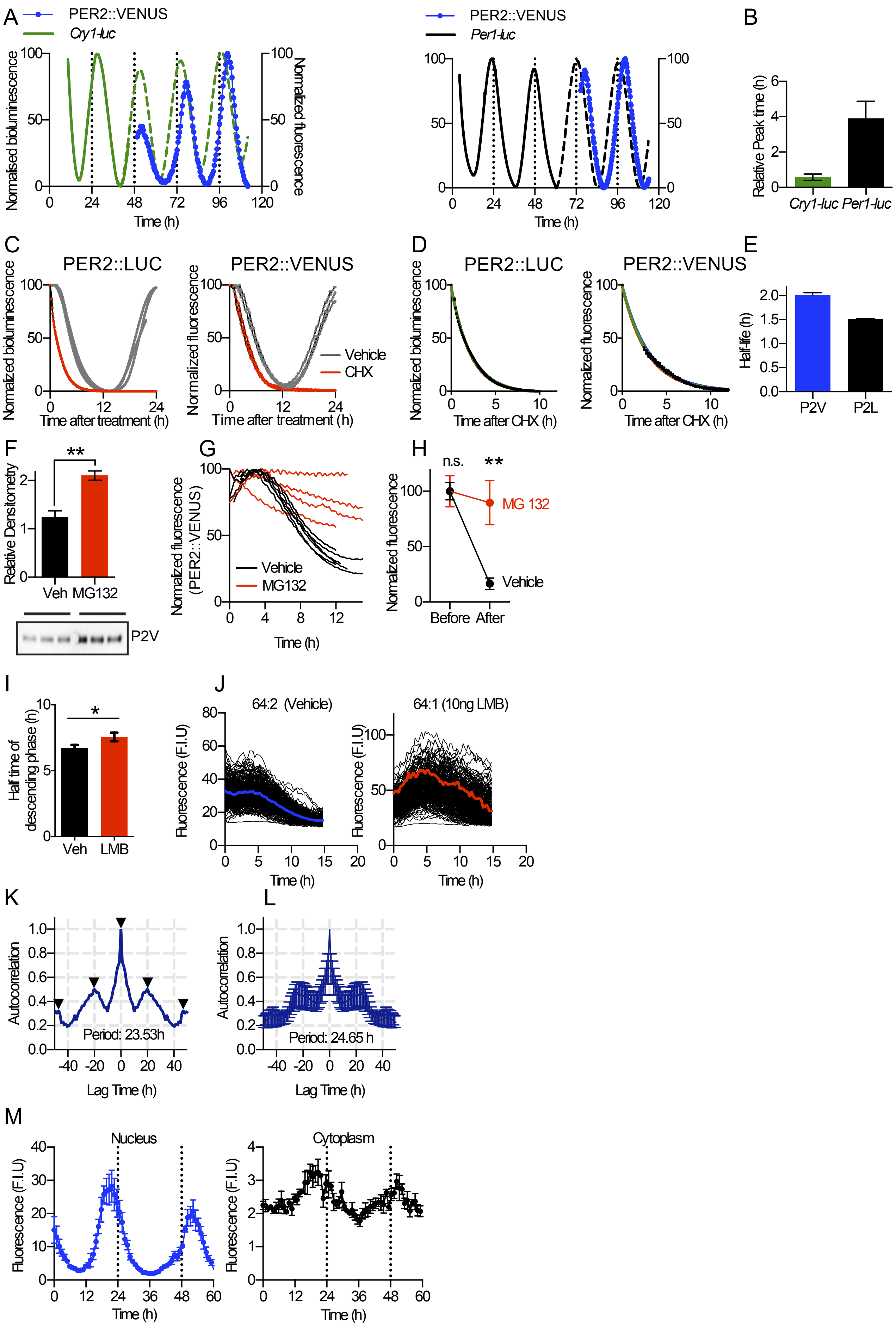

A
PER2::VENUS
Cry1-luc
Normalised bioluminescence
Normalized fluorescence
Time (h)
PER2::VENUS
Per1-luc
B
Relative Peak time (h)
Cry1-luc
Per1-luc
C
PER2::LUC
PER2::VENUS
Normalized bioluminescence
Normalized fluorescence
Time after treatment (h)
Vehicle
CHX
D
PER2::LUC
PER2::VENUS
Time after CHX (h)
E
Half-life (h)
P2V
P2L
F
**
Relative Densitometry
Veh
MG132
P2V
G
Normalized fluorescence (PER2::VENUS)
Vehicle
MG132
Time (h)
H
n.s.
**
MG 132
Vehicle
Normalized fluorescence
Before
After
I
*
Half time of descending phase (h)
Veh
LMB
J
64:2 (Vehicle)
64:1 (10ng LMB)
Fluorescence (F.I.U)
Time (h)
K
Autocorrelation
Period: 23.53h
Lag Time (h)
L
Autocorrelation
Period: 24.65 h
Lag Time (h)
M
Nucleus
Cytoplasm
Fluorescence (F.I.U)
Time (h)

Fig. S3. Probing PER2::VENUS macrodynamics using reporters and pharmacological agents. Related to Figure 3.

(A) Representative traces showing consecutive recording of *Cry1-luc* (left) or *Per1-luc* (right) reporter bioluminescence measured by PMT followed by PER2::VENUS fluorescence (blue) captured by confocal microscopy, within the same slice. Dotted lines represent extrapolated rhythms of the bioluminescence reporters. (B) Phase-mapping circadian reporters on to PER2::VENUS. Peak times (mean ± SEM) of reporter circadian rhythms relative to PER2::VENUS, quoted as time prior to PER2::VENUS peak. (C) 24 hour recordings of PER2::LUC bioluminescence and PER2::VENUS fluorescence in SCN slices (n = 3 per group) treated with cycloheximide (40 μM; red trace) or vehicle (grey) at the peak of Per2 expression. (D) Using the data shown in (C), a one-phase exponential decay was fitted to the "peak-to-trough" of traces (ca. first 10 h). (E) Half-life of PER2::LUC and PER2::VENUS (mean ± SEM). (F) Western blot and band relative density measure for PER2::VENUS fibroblasts treated with either vehicle (Veh) or 25 μM MG132. MG132 significantly increased abundance of PER2::VENUS (n_{veh} =3; n_{MG132} =3; Student's T-test, ** $p < 0.005$). (G) Mean PER2::VENUS fluorescence of individual SCN slices, treated with 50 μM MG132 (red) or vehicle (0.01% DMSO), at the peak of PER2::VENUS expression and tracked for 15 hours following treatment. (H) Fluorescence (mean ± SEM) at the peak (before treatment) and the trough (after 15 hours treatment of MG132 and vehicle treated SCN slices). A significant increase of fluorescence was observed in MG132 treated slices compared with vehicle treated (n =5, repeated-measures two-way ANOVA with Sidak's multiple comparisons test, ** $p < 0.005$). (I) Half-life (mean ± SEM) of descending phase of the cycle (peak to trough) was significantly increased by Leptomycin B (LMB) treatment (n_{veh} =5; n_{LMB} =4, Student's T-test, $p < 0.05$). (J) Single cell analysis of PER2::VENUS fluorescence in representative SCN slices treated with either vehicle or 10 LMB (ng/μL). Mean fluorescence is shown by colored lines. (K) Representative trace showing autocorrelation analysis applied to a nuclear fluorescence trace measured for 80 h, to calculate circadian period. Autocorrelation peak locations are marked with arrows. (L) Mean ± SD of autocorrelation data (n = 19). (M) Fluorescence intensity (mean ± SEM) measured in the nucleus (left panel) or cytoplasm (right panel) of individually tracked cells (n =3) across 60 hours of recording.

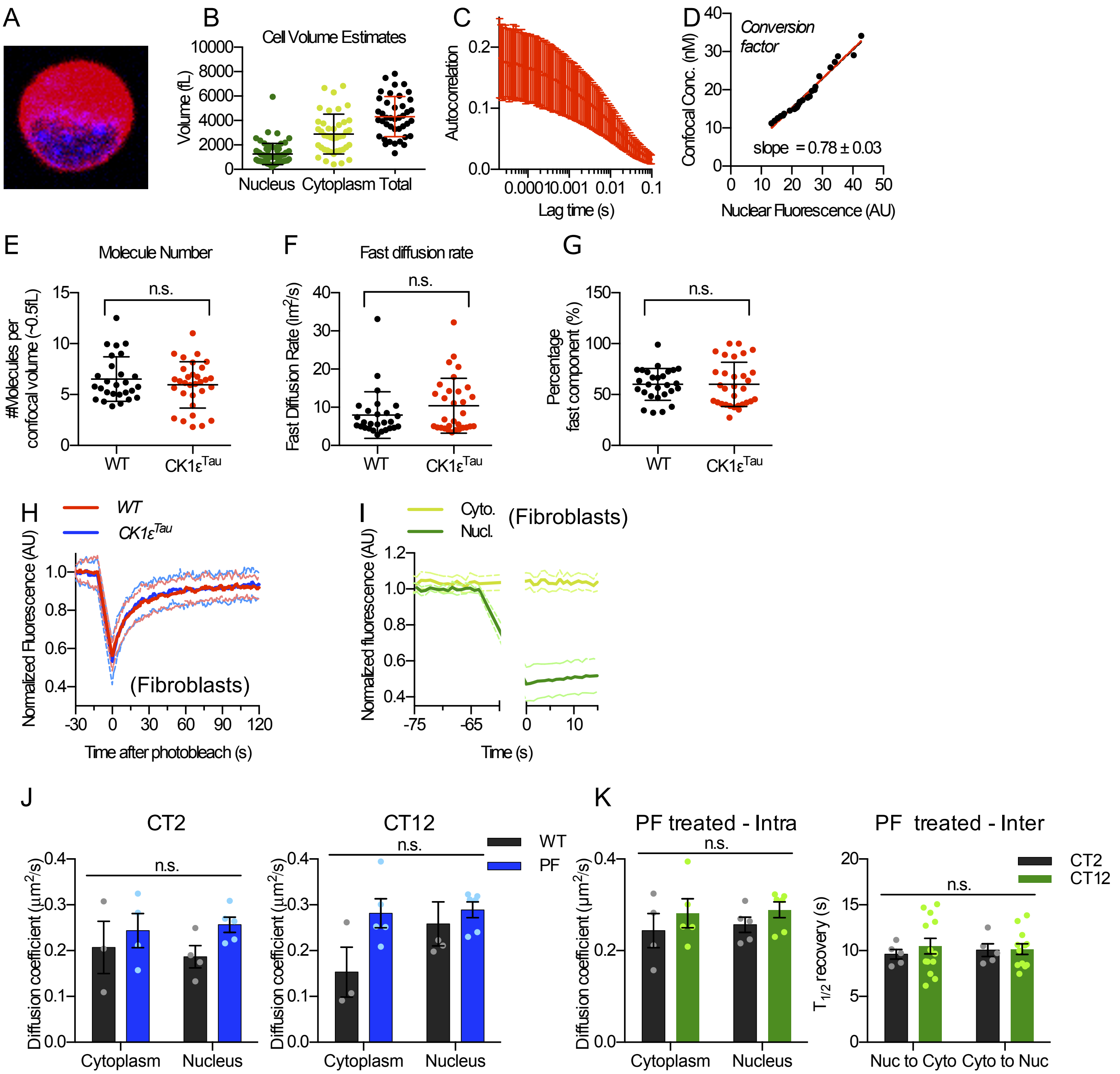
A
B
Cell Volume Estimates
Volume (fL)
10000
8000
6000
4000
2000
0
Nucleus
Cytoplasm
Total
C
Autocorrelation
0.2
0.1
0.0
0.0001
0.001
0.01
0.1
Lag time (s)
D
Conversion factor
Confocal Conc. (nM)
40
30
20
10
0
slope = 0.78 ± 0.03
0
10
20
30
40
50
Nuclear Fluorescence (AU)
E
Molecule Number
n.s.
#Molecules per confocal volume (~0.5fL)
15
10
5
0
WT
CK1ε^Tau
F
Fast diffusion rate
n.s.
Fast Diffusion Rate (im^2/s)
40
30
20
10
0
WT
CK1ε^Tau
G
n.s.
Percentage fast component (%)
150
100
50
0
WT
CK1ε^Tau
H
WT
CK1ε^Tau
Normalized Fluorescence (AU)
1.0
0.8
0.6
0.4
(Fibroblasts)
-30
0
30
60
90
120
Time after photobleach (s)
I
Cyto.
Nucl.
(Fibroblasts)
Normalized fluorescence (AU)
1.2
1.0
0.8
0.6
0.4
-75
-65
0
10
Time (s)
J
CT2
n.s.
Diffusion coefficient (µm^2/s)
0.4
0.3
0.2
0.1
0.0
Cytoplasm
Nucleus
CT12
n.s.
WT
PF
Diffusion coefficient (µm^2/s)
0.4
0.3
0.2
0.1
0.0
Cytoplasm
Nucleus
K
PF treated - Intra
n.s.
Diffusion coefficient (µm^2/s)
0.4
0.2
0.0
Cytoplasm
Nucleus
PF treated - Inter
CT2
CT12
n.s.
T1/2 recovery (s)
20
15
10
5
0
Nuc to Cyto
Cyto to Nuc

Figure S4. Quantitative measures of PER2::VENUS intracellular dynamics. Related to Figures 3 and 4. (A) Representative z-stack image of a dissociated (using trypsin) skin fibroblast. Cytoplasm is stained with CellTracker™ Red CMTPX and cell nucleus with Hoechst 33342, used to calculate cell volume. (B) Nuclear and cytoplasmic volume measurements (n =41 cells). (C) Autocorrelation curve for the peak of PER2::VENUS expression (mean ± SD, n =27). (D) Quartile-quartile plot comparison between peak confocal concentrations and mean peak nuclear fluorescence, used as a calibration for converting fluorescence intensity into PER2::VENUS concentration. (E) Number of molecules in confocal volume. The confocal volume had previously been estimated at 0.59 ± 11 fL using Rhodamine 6G. The *CK1ε ^{Tau}* allele had no effect on the numbers of PER2::VENUS molecules in the nucleus (n_{WT} =28; n_{Tau} =31; T-test). (F) "Fast" component (as defined in Fig.3E) diffusion rate was not significantly different between *WT* and *Tau* mutant fibroblasts (n_{WT} =19; n_{Tau} =25; T-test). (G) Proportion of cells displaying "fast" component is not significantly different between WT and Tau mutant fibroblasts (n_{WT} =27; n_{Tau} =31 cells; T-test). All above group data are mean ± SEM. (H) Normalized fluorescence intensity (mean ± SD) in bleached nuclear ROI (5μm diameter circle) for the recovery of photobleached WT (red, n = 19) and *CK1ε^{Tau}* (blue, n =25) temperature synchronized fibroblasts. (I) Fluorescence recovery curves (mean ± SD, blue line: n =14, red line: n =13) observed following nuclear ROI fluorescence bleaching (5μm diameter). (J) PF670462 treatment (1 uM) does not alter PER2::VENUS mobility within (intra-compartment) the cytoplasm or nucleus at CT2 (left) or CT12 (right), compared to WT (CT2: $n_{WT,cyto}$ =3; $n_{PF,cyto}$ =5; $n_{WT,nuc}$ =4; $n_{,PF,nuc}$ =6; CT12: $n_{WT,cyto}$ =4; $n_{PF,cyto}$ =5; $n_{WT,nuc}$ =5; $n_{,PF,nuc}$ =6; two-way ANOVA). (K) Intra-compartment (left) and inter-compartment (right) mobility of PER2::VENUS during PF670462 treatment. Note that same intra-compartment data are shown in separate graphs in (J) (blue bars). Intra-compartment mobility of PER2::VENUS does not change between CT, or compartment during treatment ($n_{CT2,cyto}$ =4; $n_{CT12,cyto}$ =5; $n_{CT2,nuc}$ =5; $n_{CT12,nuc}$ =6; two-way ANOVA). Inter-compartment mobility of PER2::VENUS does not change between CT, nor direction of movement Nucleus-to-cytoplasm or cytoplasm-to-nucleus) during treatment ($n_{CT2,nuc->cyto}$ =5; $n_{CT12,nuc->cyto}$ =13; $n_{CT2,cyto->nuc}$ =5; $n_{CT12,cyto->nuc}$ =12; two-way ANOVA).

Table S1. Percentage of neuropeptidergic cell type that express PER2::VENUS at different times of day. Related to Figure 1. (*n* = total number of brain sections, n_{brains} = 4)

	VIP			AVP			GRP		
ZT	*n*	Mean	SEM	*n*	Mean	SEM	*n*	Mean	SEM
0	10	0	0	10	3.0	1.4	10	0	0
12	7	99.2	0.46	10	99.3	0.58	8	7.1	3.5

Supplemental experimental procedures

Animals

All animals were cared for in accordance with the UK Animals (Scientific Procedures) Act of 1986 with local ethical approval. *CK1ε*^{Tau} mice were used as previously described [S1]. *Per1-luc* reporter mice were acquired from Hitoshi Okamura (Kyoto University, Japan) [S2]. *Per1*$^{-/-}$ mice were obtained from David Weaver (UMASS Medical School, Worcester, MA, USA) [S3].

Generation of PER2::VENUS animals

A *Venus-Neomycin* construct (Figure S1) was assembled and sent to Biocenter Oulu (University of Oulu, Finland) for generation of knock-in mice using a gene-targeting strategy in 129Sv embryonic stem (ES) cells. Resulting ES cells were injected into blastocyst stage C57Bl6 embryos. The F1 generation mice were tested for the presence of *Per2*$^{Venus\text{-}Neo}$ by Southern blot. *Neo* was removed by crossing with a cre-recombinase deleter mouse line. Mice from the F1 generation of this cross were genotyped for the presence of *Per2*^{Venus} and the loss of *Neo.* Positive mice were then crossed with C57/BL6 mice (>6 generations) and subsequently bred to give homozygosity for the *Per2*^{Venus} allele.

Primers:

P1: CTGTGTTTACTGCGAGAGT (*Per2* exon 23)

R1: GGGTCCATGTGATTAGAAAC (*Per2*^{WT})

R2: TTGAAGAAGTCGTGCTGC (*Per2*^{Venus})

Wheel-running

Mice were entrained to a 12:12 light-dark (LD - 45-50 lux with white light and 5 lux in red light) cycle for at least 10 days prior to a schedule of continuous dim red light (DD) for 14 days. Data were analyzed using ClockLab (ActiMetrix Inc., USA) running within Matlab (Mathworks, USA), as previously described [S4]. As well as circadian period, we calculated the daily activity patterns (from 3 days of DD activity data), which were then normalized, aligned and plotted as mean profiles for *WT* and *Per2*^{Venus} genotypes. Non-Parametric circadian rhythm analysis (NPCRA) was applied to 12 days ("days" were adjusted to match the circadian period of the individual animal) of wheel-running data, which generated the following parameters:

1. **Relative Amplitude (RA)** – Non-Parametric measure of rhythm amplitude between the mean lowest activity and mean highest daily activity measured over 5-hour and 10-hour time-windows, respectively.

$$RA = (Max_{average} – Low_{average}) / (Max_{average} + Low_{average})$$

2. **Intra-daily Variability (IV)** – Non-Parametric measure of frequency of activity/rest transitions. Can range between 0 (data fits Sine wave) to 2 (Gaussian noise).

3. **Inter-daily Stability (IS)** – how well daily activity patterns match on day-to-day basis. Can range from 0 (Gaussian noise) to 1 (high-stability).

These collectively give an indication of circadian coherence and robustness. Robust, rhythmic behavior would be characterized as having a high RA, low IV and high IS.

Fibroblast culture and temperature entrainment

Primary cultured lung fibroblasts from PER2::VENUS mice were seeded in DMEM/10 % FBS/Penstrep and split in a 1:2 ratio until passage 6 and then seeded into 3.5 cm dishes at a confluence ~ 300000 cells/dish. Once cells had attached, medium was changed and dishes transferred to an 5% CO_2 incubator set to alternate with 12h cycles of high (37 °C) and low (32 °C) temperature for four cycles. The samples were then transferred to constant high temperature (37 °C). For imaging, cells were processed 4-6 h after transfer to 37 °C and for Western blots, cells were harvested every four hours for 48 h. Dishes were washed in cold PBS before being frozen down at -80 °C and stored briefly before lysis and further processing. Each dish was lysed in 50 µL cold lysis buffer: 1 % NP40/ 0.25% sodium deoxycholate/ 50 mM Tris/ 1x Roche Protease Inhibitor Cocktail. The lysates were sonicated on ice for 30 seconds and a soluble protein fraction was obtained by centrifuging for 10 min at 21000 *g*. The protein lysates were normalized by BCA protein assay (Pierce, USA).

Gel electrophoresis and Western blotting

Protein lysates were subject to gel electrophoresis using NuPAGE Novex 4–12% Bis-Tris gradient gels (Life Technologies, USA), and run as per the manufacturer's protocol, using the MOPS buffer system. A wet tank system was used for protein transfer to PVDF membrane (30 V for 1 h at 4 °C). PVDF membranes were blocked for 1 hour (0.5% w/w non-fat dried milk (Marvel, USA) in TBST). Membranes were then incubated with anti-rabbit GFP antiserum (1:10000, Abcam, U.K.) overnight at 4 °C, followed by three 20 min washes. The ECL Prime (GE Healthcare, USA) chemiluminescence detection system and imaged using the GelDoc system (Biorad, USA). To check protein loading was even in the gels, duplicate gels were stained with

Coomassie InstantBlue (Expedeon, uSA). Densiometric quantification of Western blots was performed by Image Lab (Bio Rad, USA).

Immunofluorescence

PFA-fixed (4% PFA in phosphate buffer) SCN sections (40 μm) were blocked in 5% normal serum, matched to the primary antisera, in blocking buffer (BSA 1%, 0.3% Triton X-100 in PBS) for one hour, with gentle shaking. Sections were incubated with primary antisera overnight, at 4 °C, with gentle shaking. They were then washed twice for 5 minutes and incubated with secondary antiserum (Alexa 647, 1:500, Life Technologies, USA) for one hour at room temperature. Sections were then washed twice for 5 minutes in wash buffer (BSA 1%, 0.3% Triton X-100 in PBS) and then twice for 5 minutes in PBS. Sections were mounted with Vectashield with DAPI (Vectorlabs, USA), ready for confocal imaging. Primary antisera used: rabbit anti-AVP (1:1000; Bachem, USA), guinea-pig anti-VIP (1:1000, Bachem, USA) and rabbit anti-GRP (1:1000; Immunostar, USA). Both immunofluorescence and native VENUS florescence were imaged using a Zeiss 780 inverted confocal set-up (Zeiss, Germany). Low magnification images were acquired using a 20x objective and high magnification images were acquired using a 63x oil objective.

Fluorescence co-localization analysis

Semi-automated cell counts were made using the “Nucleus Counter” ImageJ (NIH, USA) plugin [S5]. Mander’s co-localisation analysis was conducted using the Mander’s Coefficient plugin within ImageJ. This analysis generates a table containing the Mander’s coefficient as well as values for M1 and M2, which distinguish the co-localisation of channel 1 with channel 2 and channel 2 with 1. For example, all of

channel 1 may co-localize with channel 2, but only 50% of channel 2 may co-localize with channel1, in which case M1 =1.0, M2 =0.5.

Organotypic slices

SCN slice cultures were made as previously described [S6] and kept in culture for 3 days prior to real-time confocal imaging, or 7 days prior to bioluminescence recordings. Whole-slice bioluminescence emissions were detected by photon multiplier tubes (Hamamatsu, Japan), set-up to count emitted photons every second in 6 minute bins.

Real-time confocal microscopy

Real-time imaging of fibroblasts and SCN slices was conducted using the Zeiss LSM780 inverted system (Zeiss, Germany), with a heated chamber kept at 37 °C. Samples were placed in air-tight, glass-bottom imaging dishes (Mattek, USA). For long-term imaging, a 10x objective was used, and images acquired 2 frames per hour (fph), 30 seconds scan time per frame, for ~70 hours. Fibroblasts were imaged for ~ 70 hours at 6 fph, with a pixel dwell time of 1.58 μs.

Drug treatments

Temperature-entrained primary lung fibroblast at peak PER2::VENUS expression (~4 h after 37˚C→32˚C) were incubated for 6 h in either 25 μM MG132 proteasome inhibitor (Cayman Chemical Company, USA) or vehicle. Lysis etc. was performed as described as above. Prior to all pharmacological treatments of $Per2^{Venus}$ SCN slices, "pre-treatment" fluorescence measures were acquired by confocal microscopy. Confocal image time-series were then acquired once drug treatments were applied to the slices, imaged at a rate of 4 frames per hour. In SCN slices, cycloheximide (Sigma Aldrich, USA) was used at 40 μM,

leptomycin B (Sigma Aldrich, USA) at 10 ng/mL and MG132 at 50 μM. Imaging data were either analyzed using mean fluorescence of the whole SCN or single cell fluorescence from the slice. The latter employed the SARFIA package [S7] running within Igor Pro (Wavemetrics, USA) to select "cell-like" regions of interest.

Fluorescence Correlation Spectroscopy and Quantified Time-lapse Imaging

FCS and quantified time-lapse were conducted as previously described [S8], using a Zeiss LSM780 on an Axio observer Z1 microscope using a plan-apochromat 63x NA 1.4. VENUS fluorescence was excited by 514 nm laser light and emission collected between 518 and 535nm after passing through a pinhole set to one Airy unit. Laser power was typically <5% total power, but was adjusted as necessary to avoid photo-bleaching and also to give a suitable count rate with a minimum 1kHz counts per molecule. The protocols as outlined in Kim et al. [S9] were followed with 10x10s runs used for each measurement. Zen 2010B software was used for data collection. A custom made script for data fitting was written in MATLAB R2014b using the Optimisation Toolbox based on the Marquardt-Levenberg algorithm. The distribution of FCS-measured peak concentration was also used to calibrate confocal data sets by quantile–quantile matching to a respective distribution of peak fluorescence as determined by image analysis.

Fluorescence recovery After Photobleaching (FRAP)

SCN slices were cut from their membrane insert on which they had been cultured, and positioned facedown on to the imaging glass to bring the tissue within the working distance of the 40x objective.

FRAP was conducted with a Zeiss LSM780, using a 40x water immersion objective. SCN slices used for FRAP experiments were either with or without casein kinase 1 inhibitor (PF670462) at 1 μM. FRAP measurements were taken at either CT2 or CT12. Four different types of photobleach were used: whole nucleus, 3 μm diameter disc in the nucleus (part nucleus), whole cytoplasm, 3 μm diameter disc in the cytoplasm (part cytoplasm). For each, the following protocol was used: 10 consecutive pre-bleach images acquired at low laser intensity (10%), followed by 100 times repeat exposure to photo-bleach at 100% laser intensity, and post-bleach image acquisition at 10 frames per second.

FRAP data analysis

FRAP data were processed to remove background noise, corrected for acquisition bleaching and normalized to 100%. The $t_{1/2}$ of recovery was calculated and diffusion coefficient estimated using a simple diffusion model [S10] given by: $D = 0.88w^2 / 4\ t_{1/2}$.

These steps are outlined below:

Mean fluorescence intensity was measured from the selected bleach area (Y_{raw}), outside the cell (Y_{bg}) and different unbleached cell (Y).

1. Background subtraction: The raw data from the bleach spot was first background subtracted (Y_{bs}) using the background measurement:

$$Y_{bs} = Y_{raw} - Y_{bg}$$

2. Calculate acquisition bleaching correction factor: Y_{pc} was fitted to a one-phase decay non-linear regression given by:

$$Y = Y_0(exp(-kt)$$

where Y is Y_{pc}, Y_0 is Y_{pc} at $t = 0$, k is the acquisition correction factor and t is time in seconds.

3. Acquisition bleaching correction: The calculated value for k was used in the following equation and applied to background subtracted data:

$$Y_{pc} = Y_{bg} + (Y_{bg,t=0} - (Y_{bg,t=0} (exp(-kt))))$$

Where Y_{pc} is photo-bleach corrected signal, Y_{bg} is background subtracted signal and $Y_{bg,t=0}$ is background subtracted signal at $t = 0$

4. Normalization: The value for Y_{pc} is normalized so that Y_{pc} at time = 0 is 100%

5. Normalized data was fit to the standard FRAP equation:

$$Y = A(1- exp(-k_2t)$$

where the value for k_2 used calculate the $t_{1/2}$ of fluorescence recovery:

$$D = 0.88w^2 / 4t_{1/2}$$

Circadian analyses of bioluminescence rhythms

Bioluminescence rhythm data generated from PMTs were analyzed using non-linear fast-Fourier transform (NL-FFT) as part of circadian analysis software running within Biodare (Prof. A. Millar, University of Edinburgh; www.biodare.ed.ac.uk). To avoid potential initialization artifacts in the analyses, the first 24 hours of the recordings were omitted. Circadian period, amplitude relative phase and relative amplitude error (RAE) were calculated for each dataset.

Statistical tests

All graphs and statistics were generated using Prism (Graphpad, USA), unless specified. Datasets containing two groups were tested by Student's two-tailed t-test. Data composed of three or more groups and a treatment variable were analyzed by two-way ANOVA with Tukey's post-hoc multiple comparison test.

Supplemental References

S1. Meng, Q.J., Logunova, L., Maywood, E.S., Gallego, M., Lebiecki, J., Brown, T.M., Sladek, M., Semikhodskii, A.S., Glossop, N.R.J., Piggins, H.D., et al. (2008). Setting clock speed in mammals: The CK1 epsilon tau mutation in mice accelerates circadian pacemakers by selectively destabilizing PERIOD proteins. Neuron *58*, 78-88.

S2. Yamaguchi, S., Mitsui, S., Miyake, S., Yan, L., Onishi, H., Yagita, K., Suzuki, M., Shibata, S., Kobayashi, M., and Okamura, H. (2000). The 5 ' upstream region of mPer1 gene contains two promoters and is responsible for circadian oscillation. Curr Biol *10*, 873-876.

S3. Bae, K., Jin, X.W., Maywood, E.S., Hastings, M.H., Reppert, S.M., and Weaver, D.R. (2001). Differential functions of mPer1, mPer2, and mPer3 in the SCN circadian clock. Neuron *30*, 525-536.

S4. Maywood, E.S., Chesham, J.E., Meng, Q.J., Nolan, P.M., Loudon, A.S.I., and Hastings, M.H. (2011). Tuning the Period of the Mammalian Circadian Clock: Additive and Independent Effects of CK1 epsilon(Tau) and Fbxl3(Afh) Mutations on Mouse Circadian Behavior and Molecular Pacemaking. J Neurosci *31*, 1539-1544.

S5. Collins, T.J. (2007). ImageJ for microscopy. Biotechniques *43*, 25-30.

S6. Hastings, M.H., Reddy, A.B., McMahon, D.G., and Maywood, E.S. (2005). Analysis of circadian mechanisms in the suprachiasmatic nucleus by transgenesis and biolistic transfection. Method Enzymol *393*, 579-592.

S7. Dorostkar, M.M., Dreosti, E., Odermatt, B., and Lagnado, L. (2010). Computational processing of optical measurements of neuronal and synaptic activity in networks. J Neurosci Meth *188*, 141-150.

S8. Bagnall, J., Boddington, C., Boyd, J., Brignall, R., Rowe, W., Jones, N.A., Schmidt, L., Spiller, D.G., White, M.R.H., and Paszek, P. (2015). Quantitative dynamic imaging of immune cell signalling using lentiviral gene transfer. Integrative Biology *7*, 713-725.

S9. Kim, S.A., Heinze, K.G., and Schwille, P. (2007). Fluorescence correlation spectroscopy in living cells. Nature methods *4*, 963-973.

S10. Axelrod, D., Koppel, D.E., Schlessinger, J., Elson, E., and Webb, W.W. (1976). Mobility Measurement by Analysis of Fluorescence Photobleaching Recovery Kinetics. Biophys J *16*, 1055-1069.
