# Supplementary figures and images for: Visualizing and Quantifying Intracellular Behavior and Abundance of the Core Circadian Clock Protein PERIOD2
Source: Curr Biol. 2016 Jul 25;26(14):1880–6. doi: 10.1016/j.cub.2016.05.018 (PMC4963210; doi:10.1016/j.cub.2016.05.018)

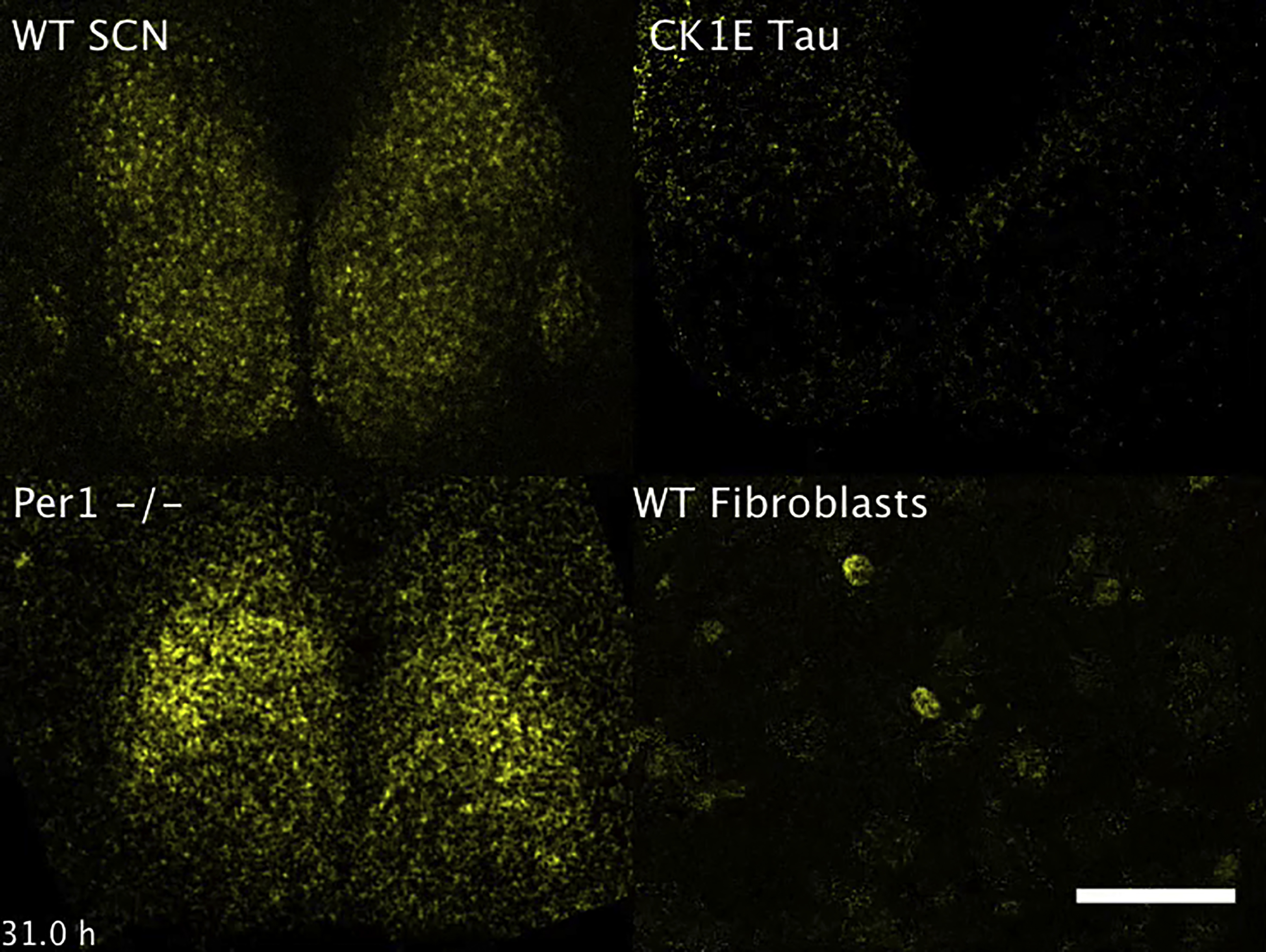

Supplement: Movie S1. PER2::VENUS Rhythms in SCN Slices and Skin Fibroblasts, Related to Figure 1 — Representative time-lapse recordings of PER2::VENUS fluorescence, recorded by confocal microscopy. Scale bar, 100 μm. [file mmc2.jpg]

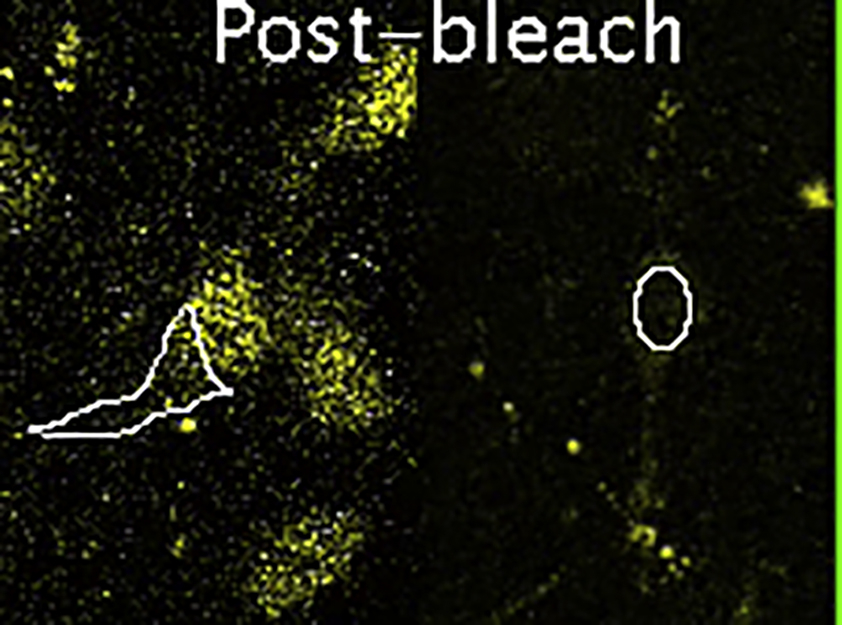

Supplement: Movie S2. FRAP after Cytoplasmic or Nuclear Photobleaching, Related to Figure 4 — Left: cytoplasmic photbleaching. Right: nuclear photobleaching. Representative time-lapse recordings represent 60 s of fluorescence recovery. Location of bleach is indicated by white outline. [file mmc3.jpg]
