# Supplementary material for: Visualizing and Quantifying Intracellular Behavior and Abundance of the Core Circadian Clock Protein PERIOD2
Source: Curr Biol. 2016 Jul 25;26(14):1880–6. doi: 10.1016/j.cub.2016.05.018 (PMC4963210; doi:10.1016/j.cub.2016.05.018)
Supplement: Document S2. Article plus Supplemental Information [file mmc4.pdf]

Report

Current Biology

Visualizing and Quantifying Intracellular Behavior and Abundance of the Core Circadian Clock Protein PERIOD2

Graphical Abstract

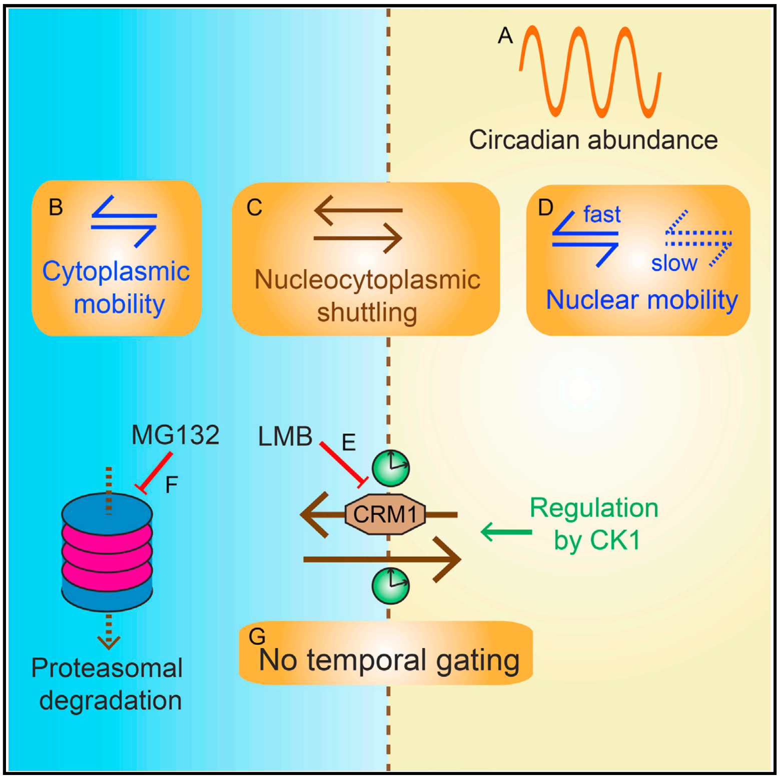

Authors

Nicola J. Smyllie, Violetta Pilorz, James Boyd, ..., Michael R.H. White, Michael H. Hastings, Andrew S.I. Loudon

Correspondence

mha@mrc-lmb.cam.ac.uk (M.H.H.), andrew.loudon@manchester.ac.uk (A.S.I.L.)

In Brief

Smyllie et al. use a fluorescent reporter mouse to image the circadian dynamics of PER2, a key component of the circadian clock in the suprachiasmatic nucleus and fibroblasts. They reveal marked divergence of the mechanisms of mouse and fly clock cells, and they provide quantitative data to support reappraisal of current models of the mammalian clock.

Highlights

- Reporter mouse is used for real-time fluorescent imaging of mammalian clock protein PER2
- In contrast to *Drosophila*, localization of PER2 is not subject to circadian gating
- Circadian abundance, mobility, and intracellular dynamics of native PER2 are quantified
- Casein kinase1 controls nucleocytoplasmic mobility of PER2 alongside circadian period

Smyllie et al., 2016, Current Biology *26*, 1880–1886
July 25, 2016 © 2016 The Authors. Published by Elsevier Ltd.
http://dx.doi.org/10.1016/j.cub.2016.05.018

Current Biology
Report

Visualizing and Quantifying Intracellular Behavior and Abundance of the Core Circadian Clock Protein PERIOD2

Nicola J. Smyllie,[1,3] Violetta Pilorz,[2,3,4] James Boyd,[2] Qing-Jun Meng,[2] Ben Saer,[2] Johanna E. Chesham,[1] Elizabeth S. Maywood,[1] Toke P. Krogager,[1] David G. Spiller,[2] Raymond Boot-Handford,[2] Michael R.H. White,[2] Michael H. Hastings,[1,*] and Andrew S.I. Loudon[2,*]
[1]Neurobiology Division, Medical Research Council (MRC) Laboratory of Molecular Biology (LMB), Francis Crick Avenue, Cambridge CB2 0QH, UK
[2]Faculty of Life Sciences, University of Manchester, Oxford Road, Manchester M13 9PT, UK
[3]Co-first author
[4]Present address: Sleep and Circadian Neuroscience Institute, Nuffield Department of Clinical Neurosciences, University of Oxford, South Parks Road, Oxford, OX1 3RE, UK
*Correspondence: mha@mrc-lmb.cam.ac.uk (M.H.H.), andrew.loudon@manchester.ac.uk (A.S.I.L.)
http://dx.doi.org/10.1016/j.cub.2016.05.018

SUMMARY

Transcriptional-translational feedback loops (TTFLs) are a conserved molecular motif of circadian clocks. The principal clock in mammals is the suprachiasmatic nucleus (SCN) of the hypothalamus. In SCN neurons, auto-regulatory feedback on core clock genes *Period* (*Per*) and *Cryptochrome* (*Cry*) following nuclear entry of their protein products is the basis of circadian oscillation [1, 2]. In *Drosophila* clock neurons, the movement of dPer into the nucleus is subject to a circadian gate that generates a delay in the TTFL, and this delay is thought to be critical for oscillation [3, 4]. Analysis of the *Drosophila* clock has strongly influenced models of the mammalian clock, and such models typically infer complex spatiotemporal, intracellular behaviors of mammalian clock proteins. There are, however, no direct measures of the intracellular behavior of endogenous circadian proteins to support this: dynamic analyses have been limited and often have no circadian dimension [5–7]. We therefore generated a knockin mouse expressing a fluorescent fusion of native PER2 protein (PER2::VENUS) for live imaging. PER2::VENUS recapitulates the circadian functions of wild-type PER2 and, importantly, the behavior of PER2::VENUS runs counter to the *Drosophila* model: it does not exhibit circadian gating of nuclear entry. Using fluorescent imaging of PER2::VENUS, we acquired the first measures of mobility, molecular concentration, and localization of an endogenous circadian protein in individual mammalian cells, and we showed how the mobility and nuclear translocation of PER2 are regulated by casein kinase. These results provide new qualitative and quantitative insights into the cellular mechanism of the mammalian circadian clock.

RESULTS

Generation and Validation of PERIOD2::VENUS Mouse

We used homologous recombination to knock in a fluorescent tag at the *Per2* locus, an equivalent strategy to that used for the PER2::LUC mouse, which is known to exhibit wild-type (WT) PER2 behavior [8]. Venus was fused to exons 19–23 of *mPer2* (Figure S1A). The presence of PER2::VENUS protein expression was confirmed by fluorescence microscopy in the brain and in lung fibroblasts (Figures 1A and 1B). As well as strong fluorescence in the suprachiasmatic nucleus (SCN), limited expression was observed in the piriform cortex, thalamus, and hippocampus (Figure S1B). Importantly, the spatial distribution of PER2::VENUS co-localized completely with PER2 immunoreactivity (-ir) in $Per2^{WT/Venus}$ SCN (Figures S1C–S1E).

To test for normal circadian function in $Per2^{Venus/Venus}$ animals, we first assessed wheel-running behavior. They entrained effectively to a 12-hr light/12-hr dark schedule (12:12 LD), and they exhibited consolidated circadian activity patterns of wheel-running when placed in constant conditions (Figures 1C, S1F, and S1G). There were no significant differences between WT and $Per2^{Venus}$ mice in the distribution, structure, or robustness (measured by non-parametric circadian rhythm analysis) of circadian behavior. After crossing with *Per1-luc* reporter mice, $Per2^{Venus/Venus}$ SCN organotypic slices expressed robust, high-amplitude circadian bioluminescence rhythms (Figures 1D and S1H). The circadian periods of behavioral and SCN rhythms were not significantly different between WT and $Per2^{Venus/Venus}$ mice (Figures 1E and 1F). Thus, PER2::VENUS did not compromise molecular pacemaking in the SCN or effective circadian control over behavior. To confirm that $Per2^{Venus}$ did not encode a loss-of-function mutation, $Per2^{Venus}$ mice were crossed to $Per1^{-/-}$ mice. In the absence of PER1, WT PER2 is a necessary and sufficient component of the circadian pacemaker [9]. $Per2^{Venus/Venus}$, $Per1^{-/-}$ mice exhibited robust and sustained wheel-running and SCN bioluminescence rhythms (Figures S1I and S1J), with comparable periods to $Per2^{WT/WT}$, $Per1^{-/-}$ mice (Figure S1K). Thus, $Per2^{Venus}$ encodes a functional allele of

 © 2016 The Authors. Published by Elsevier Ltd.
This is an open access article under the CC BY license (http://creativecommons.org/licenses/by/4.0/).

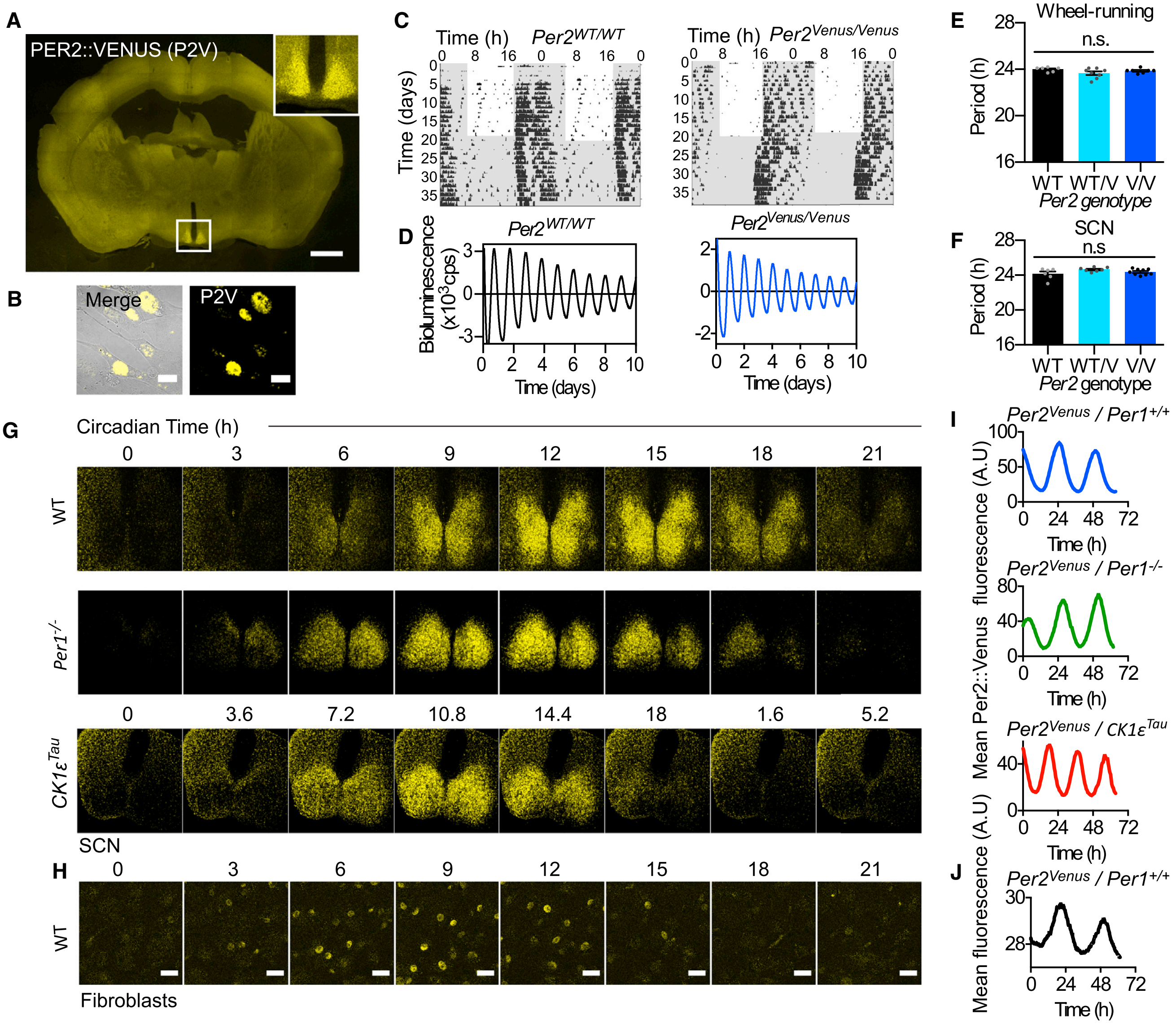

Figure 1. PER2::VENUS Fusion Protein Is a Competent Circadian Clock Protein Suitable for Real-Time Imaging

(A) PER2::VENUS fluorescence across the mouse brain, at the peak time of SCN expression (ZT12). Inset shows a close up of the SCN. Scale bar, 1 mm.

(B) Bright-field and fluorescence confocal images show $Per2^{Venus}$ lung fibroblasts. Scale bar, 20 μm.

(C) Representative, double-plotted wheel-running actograms for $Per2^{WT}$ (left) and $Per2^{Venus}$ (right) animals. Mice were entrained on a 12:12 LD cycle, followed by a schedule of constant conditions (dim red light, represented by shaded gray).

(D) Representative, de-trended *Per1-luc* bioluminescence rhythms of SCN slices from $Per2^{WT}$ (left) and $Per2^{Venus}$ (right) mice are shown.

(E) Mean ± SEM circadian periods for wheel-running are shown (n_{WT} = 6; $n_{WT/V}$ = 8; $n_{V/V}$ = 7).

(F) Mean ± SEM circadian periods for SCN slices (n_{WT} = 6; $n_{WT/V}$ = 7; $n_{V/V}$ = 9). One-way ANOVA revealed no significant effect for either measure.

(G) Snapshots from confocal real-time imaging show PER2::VENUS fluorescence in representative $Per2^{Venus}$ (top panel), Per1 null (middle panel), and $CK1\varepsilon^{Tau}$ (lower panel) in SCN slices.

(H) Snapshots from confocal real-time imaging show PER2::VENUS in fibroblasts. Scale bar, 20 μm.

(I) Mean fluorescence measures from recordings in (G) are shown.

(J) Mean fluorescence measures from recordings in (H) are shown.

See also Figure S1, Table S1, and Movie S1.

PER2. $Per2^{Venus}$ mice were then crossed with $CK1\varepsilon^{Tau}$ mutants to test whether PER2::VENUS can interact with CK1ε, a key modulator of PER2 stability and circadian period [10]. In WT animals, the $CK1\varepsilon^{Tau/Tau}$ mutation shortened period from ~24 to ~20 hr (Figures S1I, S1J, and S1L) [10]. $Per2^{Venus/Venus}$ mice showed comparable acceleration of SCN and behavioral rhythms. Thus, $Per2^{Venus}$ encodes an endogenous fusion protein that functions competently within the mammalian clock.

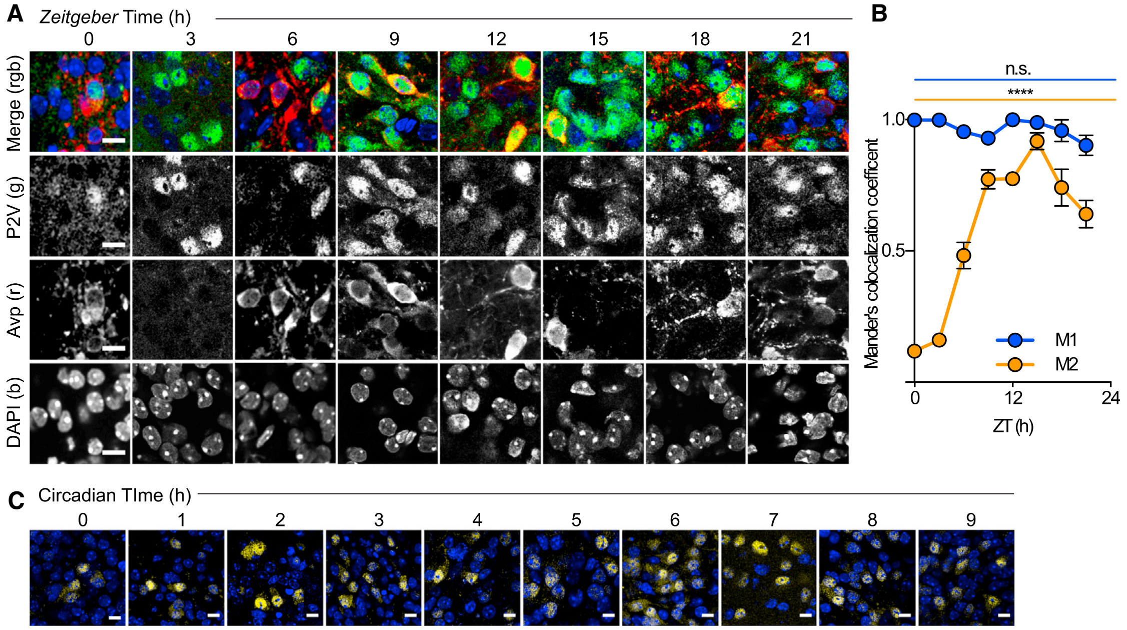

Figure 2. Circadian Subcellular Localization of PER2::VENUS

(A) Representative confocal images of SCN neurons from brain sections taken from *Per2*^{Venus} animals across the LD cycle. PER2::VENUS -positive (green) localization was compared with cytoplasmic immunostaining for AVP (red) and nuclear staining of DAPI (blue). Scale bar, 20 μm.

(B) Co-localization of PER2::VENUS and DAPI assessed by Mander's coefficient analysis (M1, blue, co-localization of PER2::VENUS with DAPI; M2, yellow, co-localization of DAPI with PER2::VENUS). Note that M2 changes across the day because the overall level of PER2::VENUS changes; thus, the proportion of DAPI nuclei containing PER2::VENUS changes.

(C) *Per2*^{Venus} SCN slices were fixed, counterstained with DAPI (blue), and imaged at different time points during the rising phase (CT0–CT9) of the PER2 circadian cycle. Each image shows representative neurons found at that time point, rather than a representative field of view at that time point.

See also Figure S2.

Intracellular Circadian Dynamics of Endogenous PERIOD2

We next analyzed the rhythmicity of the PER2::VENUS protein. A clear circadian oscillation of PER2::VENUS abundance was detected by western blot in temperature-entrained lung fibroblasts (Figures S1M and S1N). PER2::VENUS was also highly and rhythmically expressed in the SCN (Figures S1O and S1P). At the peak of PER2 expression (zeitgeber time 12 [ZT12]), in the SCN, PER2::VENUS was detected in effectively all arginine vasopressin (AVP)-immunoreactive (ir) and vasoactive intestinal peptide (VIP)-ir neurons but in <10% of gastrin-releasing peptide (GRP)-ir neurons (Figure S2; Table S1). At the trough of the cycle (CT0), only a few AVP-ir cells expressed PER2::VENUS (Table S1). We next tested the utility of PER2::VENUS as a real-time circadian reporter, using confocal microscopy. Both SCN slices and lung fibroblasts exhibited stable, high-amplitude circadian oscillations of fluorescence throughout 70 hr of recording (Figures 1G and 1H; Movie S1). In the SCN, PER2::VENUS peaked appropriately at 1 and 4 hr, respectively, after *Cry1-luc* and *Per1-luc* (Figures S3A and S3B). Circadian expression of PER2::VENUS was well defined in the SCN with a *Per1*$^{-/-}$ background and accurately reported period shortening by the *CK1ε*^{Tau} mutation. Thus, PER2::VENUS is a high-fidelity real-time reporter of the behavior of an endogenous clock protein in SCN neurons and fibroblasts.

We next determined the macrodynamics of PER2::VENUS. Using cycloheximide to inhibit translation in SCN slices, we revealed that PER2::VENUS has a half-life of∼2 hr, comparable to that of PER2::LUC (Figures S3C–S3E) [10]. Consistent with proteasomal degradation of Per2^{WT} [11], application of the proteasomal inhibitor MG132 at CT12 increased PER2::VENUS levels above those of vehicle-treated lung fibroblasts (Figure S3F) and SCN slices (Figures S3G and S3H). The nuclear export inhibitor leptomycin B, applied at CT12, significantly increased the half-life of PER2::VENUS, suggesting that nuclear export facilitates degradation (i.e., PER2 is subject to degradation in the cytoplasm; Figures S3I and S3J). Moreover, synchronization between cells in SCN slices was reduced following nuclear export blockade, suggesting that timely nuclear export and degradation are necessary for transcriptional-translational feedback loop (TTFL) timing.

Gated nuclear entry of Per protein is considered a pivotal feature of circadian timekeeping. We examined the subcellular localization of PER2::VENUS in the SCN at different points across the LD cycle. Although the overall abundance of PER2::VENUS changed across the cycle, its subcellular localization did not (Figure 2A). Mander's M1 coefficient indicated almost complete co-localization of Venus with nuclear DAPI signal at all phases (Figure 2B): at no point was PER2::VENUS excluded from the nucleus, with exclusively cytoplasmic localization. To discount the possibility of a transient gate for cytoplasmic retention and nuclear entry, cellular localization was monitored in living SCN slices imaged during the rising phase (CT0–CT9) of PER2 expression. Again, when detected,

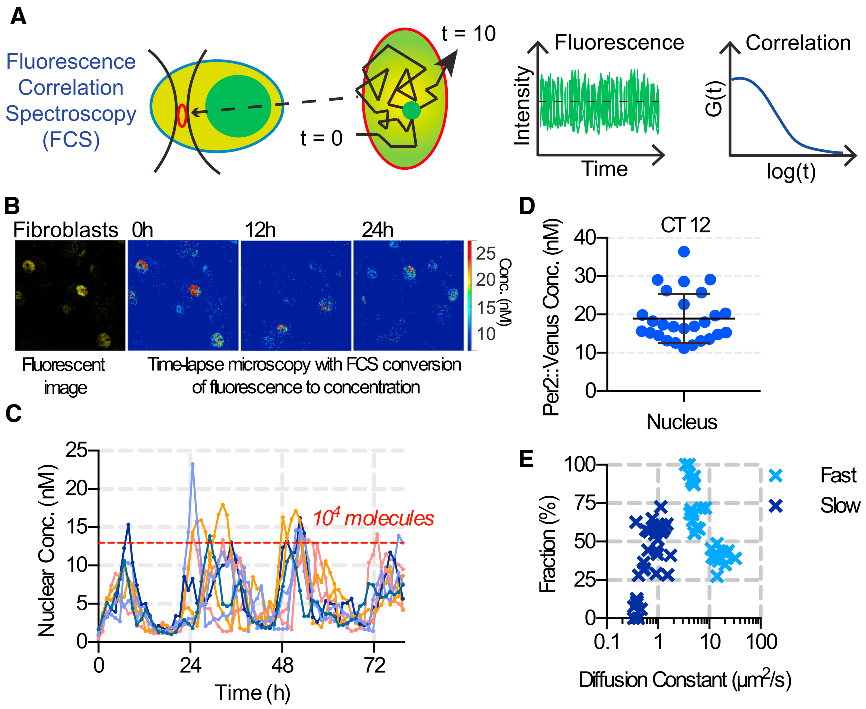

Figure 3. FCS of PER2::VENUS Protein in Fibroblasts

(A) Schematic diagram illustrating the FCS procedure. PER2::VENUS fluorescence was monitored within a small confocal volume. Individual fluorescent molecules passed through the volume at a given rate. The fluorescence signal of all of the molecules in the volume was followed through time. The concentration and rate of movement of molecules were calculated by auto-correlating the fluorescence signal.

(B) Fluorescence images and FCS-calibrated quantification of PER2::VENUS concentration in skin fibroblasts are shown.

(C) Circadian variation of nuclear concentration of PER2::VENUS over time for eight representative cells. Images were collected every 6 min but every tenth image was analyzed; thus, there is a data point every 1.12 hr.

(D) PER2::VENUS concentration in the nucleus at CT12 (4–6 hr after temperature synchronization) is shown (mean ± SEM).

(E) FCS data fit to a two-component diffusion model, color coded by component (fast, light blue; slow, dark blue), are shown.

See also Figures S3 and S4.

PER2::VENUS was observed in the nucleus at all time points (Figure 2C). This was also the case in fibroblasts, where (weak) cytoplasmic fluorescence oscillated in phase with strong nuclear fluorescence (Figures S3K–S3M). Thus, in marked contrast to the temporal gating that delays *Drosophila* Per entry to the nucleus to late subjective day (4, 5), the mouse ortholog, PER2, is not subject to compartmental circadian gating in SCN neurons or fibroblasts and nuclear accumulation occurs progressively.

Quantitative Analysis of PERIOD2 Intracellular Mobility and Abundance

Fluorescence correlation spectroscopy (FCS) was used to measure mobility and molecular abundance of PER2::VENUS in skin fibroblasts (Figure 3A). Circadian changes in PER2::VENUS concentration were observed in fibroblast nuclei (Figure 3B); but, importantly, calibration of fluorescence intensity to FCS-calculated concentration per nuclear volume enabled absolute quantification of the molecular abundance of PER2::VENUS across the circadian cycle (Figures 3C and S4A–S4D). This revealed a 10-fold amplitude cycle, with peak expression of ~15,000 molecules per nucleus (>90% of cellular total; Figure 3D). Interestingly, when FCS-derived auto-correlations were fit to a two-component diffusion model, a bimodal distribution of diffusion coefficients was determined for nuclear PER2. This indicates that it exists in at least two molecular/dynamic states, possibly as bound and unbound fractions (Figure 3E). The more mobile fraction had a diffusion coefficient of ~8 $\mu m^2 s^{-1}$. Furthermore, at CT12 the number of nuclear PER2::VENUS molecules, their diffusion rates, and the proportion displaying the slowly diffusing component were not significantly different in *CK1ε*^{Tau} fibroblasts compared to WT (Figures S3E–S3G). This suggests that PER2 mobility within the nucleus is not affected in the mutant. Thus, we have presented the first quantitative measures of intracellular dynamics of a mammalian circadian clock protein.

Fluorescence recovery after photobleaching (FRAP) was used to further examine the intracellular behavior of PER2::VENUS. Data obtained by photobleaching of fibroblast nuclei agreed with the FCS-based calculations that intranuclear PER2 mobility was unaffected by the *CK1ε*^{Tau} mutation (Figure S4H). Furthermore, after bleaching nuclear fluorescence, full recovery of nuclear fluorescence did not occur within the experimental time frame, confirming that the bulk (>90%) of PER2::VENUS was nuclear (Figure S4I). FRAP also was measured in SCN slices (Figure 4), yielding diffusion coefficients within cytoplasm or nucleus of ~0.2 $\mu m^2 s^{-1}$. This is comparable to the slow diffusing component revealed by FCS in fibroblasts (Figure 3E). The diffusion coefficient was calculated for both cytoplasm and nucleus, at CT12 and CT2. Intracompartment mobility was comparable for both cytoplasm and nucleus at both time points (Figure 4D). PER2 mobility was not reduced in the nucleus, which may have been expected for a transcriptional regulator. Translocation of PER2 into the nucleus is critical for the circadian TTFL [6, 7]. Thus, $T_{1/2}$ of FRAP was measured after photobleaching either the whole nucleus or cytoplasm (Figures 4B and 4C; Movie S2) to quantify between-compartment mobility. Importantly, this showed that there is no compartmental or temporal restriction over the mobility of PER2::VENUS (Figure 4E), in agreement with the confocal time-lapse imaging data (i.e., movement between compartments was comparable in both directions).

CK1δ/ε activity is an important regulator of the TTFL, where CK1-mediated phosphorylation of PER proteins licenses them for ubiquitination and proteasomal degradation. To test whether CK1 modulates PER2 mobility, we treated SCN slices with CK1 inhibitor PF670462, which slowed *Per1-luc* oscillations to 28.6 ± 0.30 hr (n = 10, p < 0.01 versus pre-treatment).

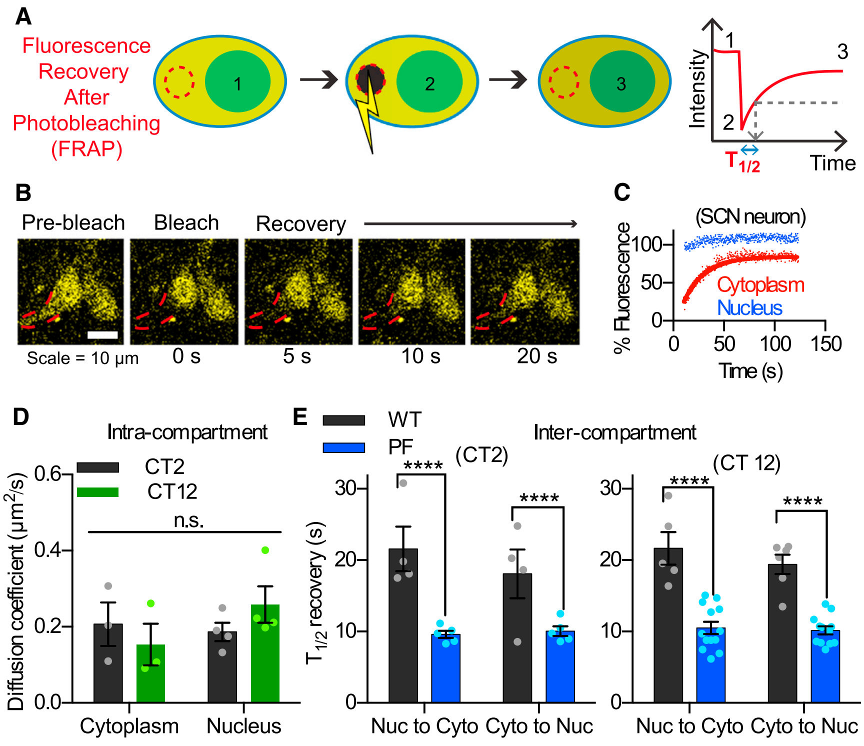

Figure 4. FRAP Reveals Role of CK1 in Regulating PER2 Mobility in SCN Neurons

(A) A schematic diagram illustrating FRAP as follows: (1) select region and measure pre-bleach fluorescence, (2) photobleach region, and (3) monitor the recovery of fluorescence. The $T_{1/2}$ is calculated from the recovery curve.

(B) Snapshots from a FRAP experiment show cytoplasmic photobleaching and fluorescence recovery in neurons in an SCN slice.

(C) Fluorescence recovery for a bleached cytoplasmic region (red) and unbleached nuclear region (blue). The latter shows no change in fluorescence over the time course.

(D) FRAP-derived diffusion coefficients (mean ± SEM) within SCN nucleus and cytoplasm were comparable at both peak and trough (CT2 and CT12) of the cycle ($n_{CT2,cyto}$ = 3 slices, $n_{CT12,cyto}$ = 4, $n_{CT2,nuc}$ = 3, and $n_{CT12,nuc}$ = 4; two-way ANOVA). Diffusion coefficients were estimated from FRAP-derived $T_{1/2}$ measures.

(E) FRAP-derived $T_{1/2}$ (mean ± SEM) calculated from total cytoplasm (Nuc to Cyto) and total nucleus (Cyto to Nuc) FRAP of SCN neurons at CT2 (left) and CT12 (right). Treatment with PF670462 (1 µM) significantly decreased $T_{1/2}$ times for PER2::VENUS ($n_{CT2,WT,cyto}$ = 4 slices, $n_{CT2,WT,nuc}$ = 4, $n_{CT2,PF,cyto}$ = 5, $n_{CT2,PF,nuc}$ = 5, $n_{CT12,WT,cyto}$ = 5, $n_{CT12,WT,nuc}$ = 6, $n_{CT12,PF,cyto}$ = 13, and $n_{CT12,PF,nuc}$ = 12; two-way ANOVA with Tukey's comparison, ****$p < 0.0001$).

See also Figure S4 and Movie S2.

Intracompartment mobility was unaffected by treatment with PF670462 (Figures S4J and S4K); but, surprisingly, both at CT2 and CT12 it significantly decreased the $T_{1/2}$ of FRAP (Figure 4E; i.e., the rates of PER2::VENUS bi-directional translocation were accelerated). Furthermore, the magnitude of the increases in $T_{1/2}$ were consistent across time points (CT2/CT12) and direction of movement (Figure S4K). Thus, decreasing CK1 activity increased PER2::VENUS translocation rates in an unbiased manner. In conclusion, low CK1ε/δ activity, which significantly lengthened the period of the SCN TTFL, was associated with accelerated nucleo-cytoplasmic shuttling of PER2::VENUS.

DISCUSSION

The PER2::VENUS mouse enables direct qualitative and quantitative observations of the complex spatiotemporal behavior of the PER2 protein, in a physiologically relevant setting. Our results suggest a pivotal role for the dynamic balance of nuclear entry and export for the determination of circadian period.

The PER2::VENUS allele was validated as clock competent, a property shared with PER2::LUC [8, 12]. Luciferase reporter systems, however, are not suitable for measuring fast events, such as nucleo-cytoplasmic shuttling of proteins. They produce a dim signal necessitating a long integration time for detection, and their inherent requirement for luciferin substrate can generate luciferase-chemistry-dependent artifacts. Fluorescent proteins do not suffer from these problems, but there are, of course, some potential limitations, including altered stability of the endogenous protein. The half-life of PER2::VENUS was similar to PER2::LUC, suggesting that the Venus tag did not alter the stability of the PER2 protein. PER2::LUC had a slightly shorter half-life, but this is in line with published literature reporting that luciferase-tagged reporters have a shorter half-life than their endogenous counterparts [13]. The PER2::VENUS half-life may be a more accurate estimate of PER2 stability, as it is a direct measure of the protein, rather than using enzymatic luciferase activity, which is an indirect measure. Prolonged fluorescence imaging can cause both phototoxicity and photobleaching, but for PER2::VENUS we found that it was possible to image SCN slices and fibroblast cultures over at least 70 hr without loss of fluorescence or circadian competence. Although there was limited detection of cytoplasmic PER2::VENUS in fibroblasts, photobleaching of the entire cytoplasm only reduced the nuclear fluorescence by <2% at CT12; thus, it is unlikely to contribute to the overall behavior of PER2 in this cell type. Overall, PER2::VENUS is a useful and faithful reporter for monitoring PER2 dynamics over shorter and longer timescales.

In contrast to the *Drosophila* ortholog, dPer, which accumulates in the cytoplasm prior to nuclear entry [3, 4], PER2 is not subject to a circadian gate in SCN neurons and in fibroblasts. This contrasting behavior may be explained by the proteins having different hetero-dimerization partners (dTim and CRY, respectively). Our data do not preclude nuclear gating of other clock factors, but there is, so far, no evidence for this. For example, snapshot immunostaining of PER1 in SCN highlights nuclear expression and no cytoplasmic restriction [14]. Progression of the TTFL in the mammalian clock, therefore, is not dependent on gated nuclear translocation; rather, it is achieved by graded nuclear accumulation of the negative regulators.

PER2::VENUS enabled us to quantify the number of PER2 molecules per cell and determine the dynamic changes in protein mobility. At CT12, PER2::VENUS was present at a concentration of ~15–20 nM in fibroblast nuclei, equating to >10,000 molecules. Interestingly, stochastic simulations of the mammalian circadian clock predicted that stability within the virtual TTFL required >4,000 molecules [15]. Thus, our real observations are of the same order of magnitude and can inform future development of in silico models. The *Per2*^{Venus} mouse also facilitates analysis of how the intracellular behavior of PER2 directs the properties of the clock. Both FRAP and FCS revealed diffusion coefficients principally in the range of 0.1 to 1.0 $\mu m^2 s^{-1}$. This is broadly compatible with data from other dynamic transcription factor proteins [16]. FCS also identified a more mobile fraction in fibroblasts, with a coefficient of ~10 $\mu m^2 s^{-1}$. The obtained fast and slow diffusion constants (~10 and ~1 $\mu m^2 s^{-1}$), as well as free and bound fractions, are comparable to estimates made by FCS for the in vivo binding of glucocorticoid receptor [17].

Numerous studies link CK1 to the regulation of PER2 function and localization [18–20]. Both *CK1ε*^{Tau} and *hPer2*^{S662G} are mutants that have a short circadian period, with altered phosphorylation of PER2. The former exhibits rapid clearance of PER2 from the nucleus [10, 21] and the latter decreased nuclear retention [22–24]. These are two aspects of the same phenomenon. Our data demonstrate that CK1 contributes to the translocation of PER2 through the nuclear pores. Thus, CK1 is a critical regulator of PER2 mobility and circadian period, although the contribution of various types of mutation to short periods is not fully resolved [25, 26].

In conclusion, PER2::VENUS operates as a functional circadian protein, and it is sufficient to sustain timekeeping in the absence of WT Per proteins. We believe that the fundamental observations we have presented will support a significant reappraisal of the mammalian clock mechanism and provide valuable observational data on an endogenous clock protein that will inform the development of enhanced quantitative models of cellular circadian timekeeping.

SUPPLEMENTAL INFORMATION

Supplemental Information includes four figures, one table, Supplemental Experimental Procedures, and two movies and can be found with this article online at http://dx.doi.org/10.1016/j.cub.2016.05.018.

AUTHOR CONTRIBUTIONS

N.J.S. and V.P. contributed equally to this work. N.J.S. designed, performed, and analyzed all SCN-related experiments. V.P. contributed to the generation and initial behavioral validation of the mouse, with input from E.S.M., Q.-J.M., and R.B.-H. J.B. conducted the FCS experiments with input from M.R.H.W. and D.G.S. B.S. managed breeding and generated primary skin fibroblasts for FCS experiments. J.E.C. supervised breeding and conducted wheel-running experiments. T.P.K. performed western blotting experiments with PER2::VENUS fibroblasts. N.J.S., M.H.H., V.P., and A.S.I.L. wrote the manuscript with input from Q.-J.M., J.B., D.G.S., and E.S.M.

ACKNOWLEDGMENTS

All animals were cared for in accordance with the UK Animals (Scientific Procedures) Act of 1986 with local ethical approval. We thank the biomedical facilities' staffs at the MRC-LMB Ares facility and the University of Manchester, We also thank Nick Barry (MRC-LMB) for expert assistance with confocal microscopy. This work was funded by Biotechnology and Biological Sciences Research Council (BBSRC) grants (BB/E022553/1 to A.S.I.L., BB/K003097/1 to A.S.I.L. and M.R.H.W., and BB/E023223/1 to M.H.H.) and the MRC (MC_U105170643 to M.H.H. and MR/K015885/1 A.S.I.L. and M.R.H.W.).

Received: March 18, 2016
Revised: May 3, 2016
Accepted: May 5, 2016
Published: June 30, 2016

REFERENCES

1. Shearman, L.P., Sriram, S., Weaver, D.R., Maywood, E.S., Chaves, I., Zheng, B., Kume, K., Lee, C.C., van der Horst, G.T.J., Hastings, M.H., and Reppert, S.M. (2000). Interacting molecular loops in the mammalian circadian clock. Science *288*, 1013–1019.
2. Koike, N., Yoo, S.H., Huang, H.C., Kumar, V., Lee, C., Kim, T.K., and Takahashi, J.S. (2012). Transcriptional architecture and chromatin landscape of the core circadian clock in mammals. Science *338*, 349–354.
3. Meyer, P., Saez, L., and Young, M.W. (2006). PER-TIM interactions in living *Drosophila* cells: an interval timer for the circadian clock. Science *311*, 226–229.
4. Shafer, O.T., Rosbash, M., and Truman, J.W. (2002). Sequential nuclear accumulation of the clock proteins period and timeless in the pacemaker neurons of *Drosophila* melanogaster. J. Neurosci. *22*, 5946–5954.
5. Tamanini, F., Yagita, K., Okamura, H., and van der Horst, G.T.J. (2005). Nucleocytoplasmic shuttling of clock proteins. Methods Enzymol. *393*, 418–435.
6. Yagita, K., Tamanini, F., Yasuda, M., Hoeijmakers, J.H.J., van der Horst, G.T.J., and Okamura, H. (2002). Nucleocytoplasmic shuttling and mCRY-dependent inhibition of ubiquitylation of the mPER2 clock protein. EMBO J. *21*, 1301–1314.
7. Yagita, K., Yamaguchi, S., Tamanini, F., van Der Horst, G.T.J., Hoeijmakers, J.H.J., Yasui, A., Loros, J.J., Dunlap, J.C., and Okamura, H. (2000). Dimerization and nuclear entry of mPER proteins in mammalian cells. Genes Dev. *14*, 1353–1363.
8. Yoo, S.H., Yamazaki, S., Lowrey, P.L., Shimomura, K., Ko, C.H., Buhr, E.D., Siepka, S.M., Hong, H.K., Oh, W.J., Yoo, O.J., et al. (2004). PERIOD2:LUCIFERASE real-time reporting of circadian dynamics reveals persistent circadian oscillations in mouse peripheral tissues. Proc. Natl. Acad. Sci. USA *101*, 5339–5346.
9. Maywood, E.S., Chesham, J.E., Smyllie, N.J., and Hastings, M.H. (2014). The Tau mutation of casein kinase 1ε sets the period of the mammalian pacemaker via regulation of Period1 or Period2 clock proteins. J. Biol. Rhythms *29*, 110–118.
10. Meng, Q.J., Logunova, L., Maywood, E.S., Gallego, M., Lebiecki, J., Brown, T.M., Sládek, M., Semikhodskii, A.S., Glossop, N.R.J., Piggins, H.D., et al. (2008). Setting clock speed in mammals: the CK1 epsilon tau mutation in mice accelerates circadian pacemakers by selectively destabilizing PERIOD proteins. Neuron *58*, 78–88.
11. Eide, E.J., Woolf, M.F., Kang, H., Woolf, P., Hurst, W., Camacho, F., Vielhaber, E.L., Giovanni, A., and Virshup, D.M. (2005). Control of mammalian circadian rhythm by CKIepsilon-regulated proteasome-mediated PER2 degradation. Mol. Cell. Biol. *25*, 2795–2807.
12. Tahara, Y., Kuroda, H., Saito, K., Nakajima, Y., Kubo, Y., Ohnishi, N., Seo, Y., Otsuka, M., Fuse, Y., Ohura, Y., et al. (2012). In vivo monitoring of peripheral circadian clocks in the mouse. Curr. Biol. *22*, 1029–1034.
13. Millar, A.J., Short, S.R., Chua, N.H., and Kay, S.A. (1992). A novel circadian phenotype based on firefly luciferase expression in transgenic plants. Plant Cell *4*, 1075–1087.
14. Field, M.D., Maywood, E.S., O'Brien, J.A., Weaver, D.R., Reppert, S.M., and Hastings, M.H. (2000). Analysis of clock proteins in mouse SCN demonstrates phylogenetic divergence of the circadian clockwork and resetting mechanisms. Neuron *25*, 437–447.

15. Forger, D.B., and Peskin, C.S. (2005). Stochastic simulation of the mammalian circadian clock. Proc. Natl. Acad. Sci. USA *102*, 321–324.

16. Bagnall, J., Boddington, C., Boyd, J., Brignall, R., Rowe, W., Jones, N.A., Schmidt, L., Spiller, D.G., White, M.R., and Paszek, P. (2015). Quantitative dynamic imaging of immune cell signalling using lentiviral gene transfer. Integr. Biol. (Camb.) *7*, 713–725.

17. Stasevich, T.J., Mueller, F., Michelman-Ribeiro, A., Rosales, T., Knutson, J.R., and McNally, J.G. (2010). Cross-validating FRAP and FCS to quantify the impact of photobleaching on in vivo binding estimates. Biophys. J. *99*, 3093–3101.

18. Akashi, M., Tsuchiya, Y., Yoshino, T., and Nishida, E. (2002). Control of intracellular dynamics of mammalian period proteins by casein kinase I epsilon (CKIepsilon) and CKIdelta in cultured cells. Mol. Cell. Biol. *22*, 1693–1703.

19. Takano, A., Isojima, Y., and Nagai, K. (2004). Identification of mPer1 phosphorylation sites responsible for the nuclear entry. J. Biol. Chem. *279*, 32578–32585.

20. Vielhaber, E., Eide, E., Rivers, A., Gao, Z.H., and Virshup, D.M. (2000). Nuclear entry of the circadian regulator mPER1 is controlled by mammalian casein kinase I epsilon. Mol. Cell. Biol. *20*, 4888–4899.

21. Dey, J., Carr, A.J.F., Cagampang, F.R.A., Semikhodskii, A.S., Loudon, A.S.I., Hastings, M.H., and Maywood, E.S. (2005). The tau mutation in the Syrian hamster differentially reprograms the circadian clock in the SCN and peripheral tissues. J. Biol. Rhythms *20*, 99–110.

22. Toh, K.L., Jones, C.R., He, Y., Eide, E.J., Hinz, W.A., Virshup, D.M., Ptácek, L.J., and Fu, Y.H. (2001). An hPer2 phosphorylation site mutation in familial advanced sleep phase syndrome. Science *291*, 1040–1043.

23. Vanselow, K., Vanselow, J.T., Westermark, P.O., Reischl, S., Maier, B., Korte, T., Herrmann, A., Herzel, H., Schlosser, A., and Kramer, A. (2006). Differential effects of PER2 phosphorylation: molecular basis for the human familial advanced sleep phase syndrome (FASPS). Genes Dev. *20*, 2660–2672.

24. Xu, Y., Toh, K.L., Jones, C.R., Shin, J.Y., Fu, Y.H., and Ptácek, L.J. (2007). Modeling of a human circadian mutation yields insights into clock regulation by PER2. Cell *128*, 59–70.

25. Vanselow, K., and Kramer, A. (2007). Role of phosphorylation in the mammalian circadian clock. Cold Spring Harb. Symp. Quant. Biol. *72*, 167–176.

26. Zhou, M., Kim, J.K., Eng, G.W.L., Forger, D.B., and Virshup, D.M. (2015). A Period2 phosphoswitch regulates and temperature compensates circadian period. Mol. Cell *60*, 77–88.

Current Biology, Volume *26*

Supplemental Information

Visualizing and Quantifying

Intracellular Behavior and Abundance

# of the Core Circadian Clock Protein PERIOD2

Nicola J. Smyllie, Violetta Pilorz, James Boyd, Qing-Jun Meng, Ben Saer, Johanna E. Chesham, Elizabeth S. Maywood, Toke P. Krogager, David G. Spiller, Raymond Boot-Handford, Michael R.H. White, Michael H. Hastings, and Andrew S.I. Loudon

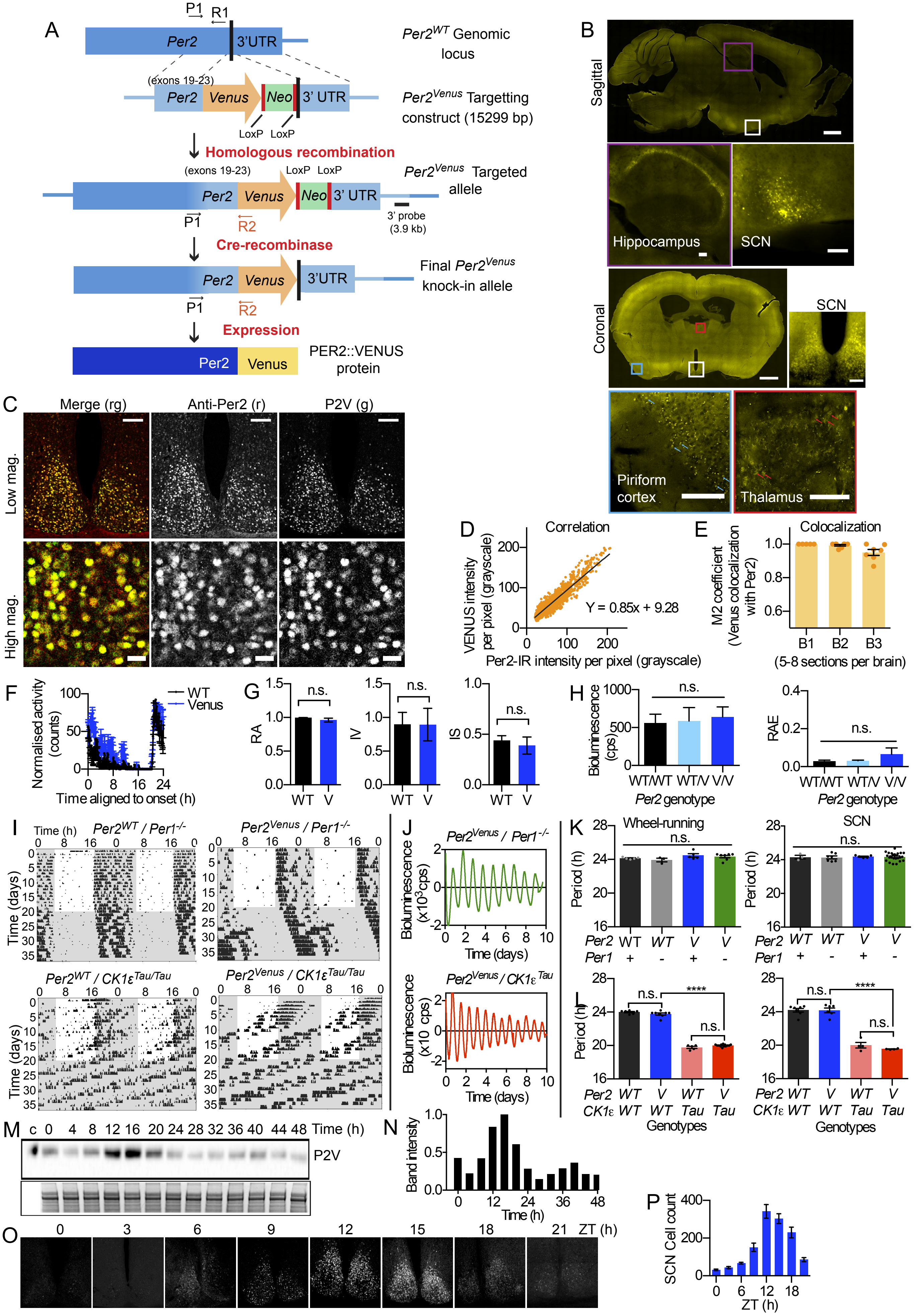
A
P1
R1
Per2
3'UTR
Per2WT Genomic locus
(exons 19-23)
Per2
Venus
Neo
3' UTR
LoxP
LoxP
Per2Venus Targetting construct (15299 bp)
Homologous recombination
(exons 19-23)
LoxP LoxP
Per2
Venus
Neo
3' UTR
Per2Venus Targeted allele
P1
R2
3' probe (3.9 kb)
Cre-recombinase
Per2
Venus
3'UTR
Final Per2Venus knock-in allele
P1
R2
Expression
Per2
Venus
PER2::VENUS protein
B
Sagittal
Hippocampus
SCN
Coronal
SCN
Piriform cortex
Thalamus
C
Merge (rg)
Anti-Per2 (r)
P2V (g)
Low mag.
High mag.
D
Correlation
VENUS intensity per pixel (grayscale)
Y = 0.85x + 9.28
Per2-IR intensity per pixel (grayscale)
E
Colocalization
M2 coefficient (Venus colocalization with Per2)
B1
B2
B3
(5-8 sections per brain)
F
Normalised activity (counts)
WT
Venus
Time aligned to onset (h)
G
n.s.
RA
IV
IS
WT
V
H
Bioluminescence (cps)
RAE
WT/WT
WT/V
V/V
Per2 genotype
I
Time (h)
Per2WT / Per1-/-
Per2Venus / Per1-/-
Time (days)
Per2WT / CK1εTau/Tau
Per2Venus / CK1εTau/Tau
J
Per2Venus / Per1-/-
Bioluminescence (x10³cps)
Time (days)
Per2Venus / CK1εTau
Bioluminescence (x10 cps)
K
Wheel-running
SCN
Period (h)
Per2
Per1
L
****
CK1ε
Tau
Genotypes
M
c
Time (h)
P2V
N
Band intensity
Time (h)
O
ZT (h)
P
SCN Cell count
ZT (h)

Fig. S1. *Per2*^{Venus} mice express a functional fusion protein that is sufficient to drive circadian rhythms in gene expression and behavior. Related to Figure 1.

(A) A schematic diagram depicting the generation of the PER2::VENUS protein. The targetting construct contains exons 19-23 of *Per2* followed by the sequence for Venus, with floxed *neomycin* as a selection marker. The targeting construct was inserted into the *Per2* locus in the mouse by homologous recombination, where the 3′ end of the endogenous gene was replaced with the targeting construct. *Neomycin* was later removed using Cre-recombinase. The final *Per2*^{Venus} knock-in allele resulted in the expression of the PER2::VENUS (P2V) fusion protein. Locations of genotyping primers are marked. P1+R1 gave a positive reaction in *Per2*^{WT} animals, whereas P1 and R2 gave a positive reaction in *Per2*^{Venus} animals. (B) Representative epifluorescence images showing PER2::VENUS expresson across the brain in both sagittal and coronal views. Snapshots of regions containing signficant PER2::VENUS expression are shown adjacent to the overview images. Their locations are marked with colored boxes. Fluorescence levels were particularly low in the piriform cortex and thalamus, so arrows highlighting cells have been included on the figure. (C) Representative confocal images showing the complete co-localisation of PER2::VENUS (green) with Per2-immunoreactivity (red) in SCN from *Per2*$^{WT/Venus}$, thus animals expressing both native *Per2*^{WT} and the *Per2*^{Venus} alleles. Upper panel shows the entire SCN (scale bar =100 µm) and lower panel shows close-up of individual cells (scale bar =20 µm). PER2::VENUS fluorescence intensity was highly correlated to Per2 immunostaining. (D) A representative correlation plot of pixel intensity in the red (Per2-IR) and green (PER2::VENUS) channels. (E) Mander's colocalization analysis indicated almost complete co-localization between the two channels (n =3). (F) Normalized, mean ± SEM daily DD wheel-running activity profile of WT ($n_{WT/WT}$ =5) and *Per2*$^{Venus/Venus}$ ($n_{V/V}$ =5) animals.

(G) Non-parametric Circadian Rhythm Analysis (NPCRA) of 12 days of DD wheel-running data. Relative Amplitude (RA; left), Intradaily Variability (IV; centre) and Interdaily Stability (IS, right) were not significantly different between WT and *Per2^{Venus}* (V) animals ($n_{WT/WT}$ =5; $n_{V/V}$ =5; unpaired T-test), thus circadian robustness of wheel-running activity was not affected by the presence of the *Per2^{Venus}* allele. (H) Circadian parameters of ampitude (left) and Relative Amplitude Error (RAE) (right) of SCN slices were not significantly affected by the *Per2^{Venus}* allele ($n_{WT/WT}$ =5; $n_{WT/V}$ =8; $n_{V/V}$ =9; one-way ANOVA). (I) Representative double-plotted actograms of wheel-running activity for *Per1$^{-/-}$* (upper) and *CK1ε$^{Tau/Tau}$* (lower) animals . Shaded areas mark times when lights are off. (J) Representative, de-trended Per1-luc bioluminescence rhythms from SCN slices. (K) In the absence of *Per1* expression (*Per1$^{-/-}$*; P1KO), SCN circadian period showed no significant effect of carrying the *Per2^{Venus}* allele (Behavior: $n_{WT,WT}$ =5; $n_{V,WT}$ =7; $n_{WT,P1KO}$ =3; $n_{V,P1KO}$ =7; SCN: $n_{WT,WT}$ =3; $n_{V,WT}$ =5; $n_{WT,P1KO}$ =7; $n_{V,P1KO}$ =24; two-way ANOVA). (L) Both wheel-running behavior (left) and SCN slices (right) from Per2$^{Venus/Venus}$ animals reported the short-period mutant phenotype of the *CK1ε$^{Tau/Tau}$* (Tau) allele (Behavior: $n_{WT,WT}$ =6; $n_{V,WT}$ =9; $n_{WT,Tau}$ =5; $n_{V,Tau}$ =13; SCN: $n_{WT,WT}$ =7; $n_{V,WT}$ =6; $n_{WT,Tau}$ =4; $n_{V,Tau}$ =4; two-way ANOVA with Tukey's comparisons: **** p <0.0001). All bar charts show mean ± SEM of group data. (M) Western blot showing rhythms of PER2::VENUS abundance in temperature-entrained *Per2$^{Venus/Venus}$* fibroblasts. (N) Band intensity measures for western blot shown in (M). (O) Representative images of fluorescence in SCN sections across the day. (P) Mean ± SEM counts of PER2::VENUS-positive cells from SCN sections taken from mice at different time points across the light-dark cycle.

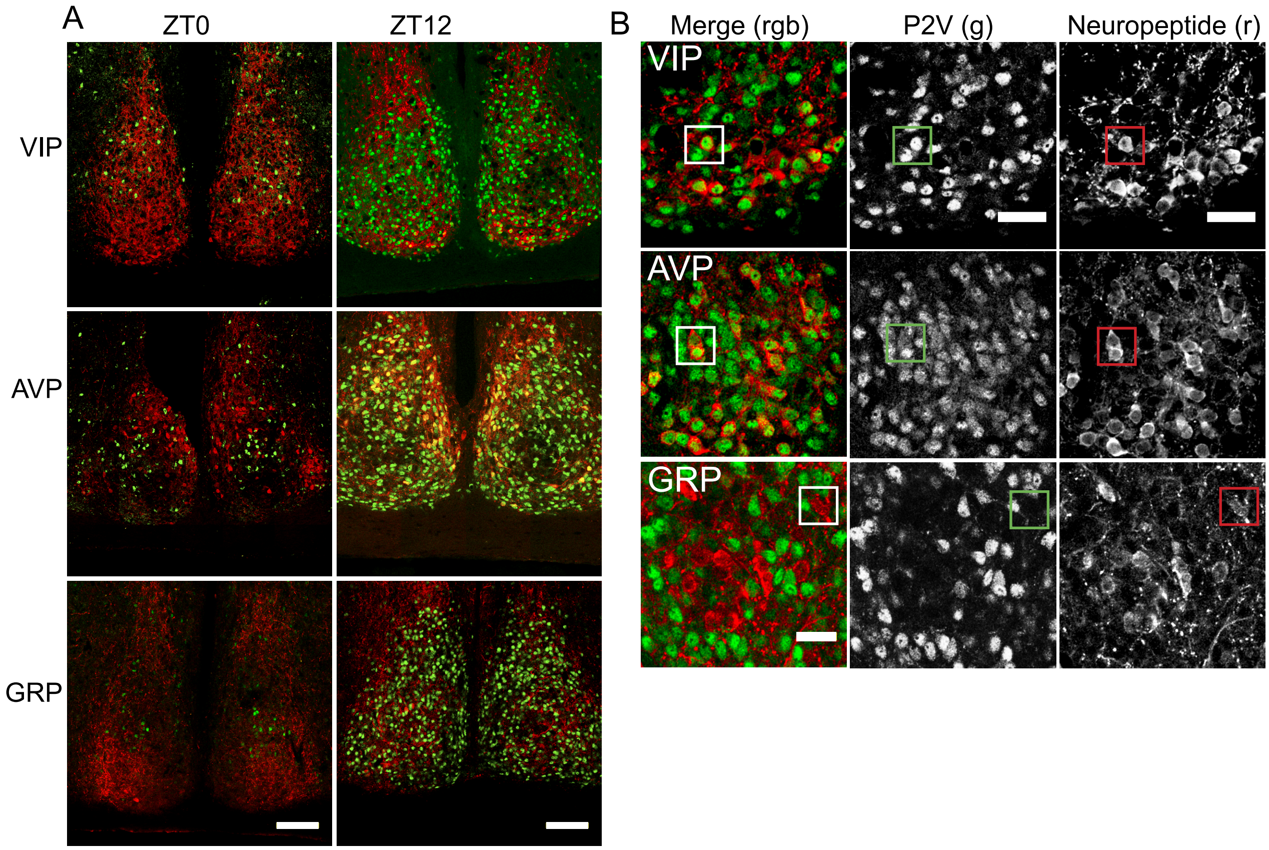

Fig. S2. Neurochemical identities of PER2::VENUS positive cells. Related to Figure 2. Representative images of SCN brain sections, showing immunofluorescence of SCN neuropeptides (red) vasoactive intestinal peptide (VIP), arginine vasopressin (AVP) and gastrin releasing peptide (GRP), colocalized with PER2::VENUS fluorescence (green) and counterstained with DAPI (blue). (A) Low-power images showing the whole SCN sections taken at ZT0 (left) and ZT12 (right). Scale bar = 100 uM. (B) Images showing "close-up" view of neuropeptide colocalization in the SCN at ZT12. The boxes (white for merged, red for neuropeptide and bue for DAPI channels), draw attention to positive colocalization of PER2::VENUS with VIP and AVP, but absence with GRP. Scale bar = 50 uM.

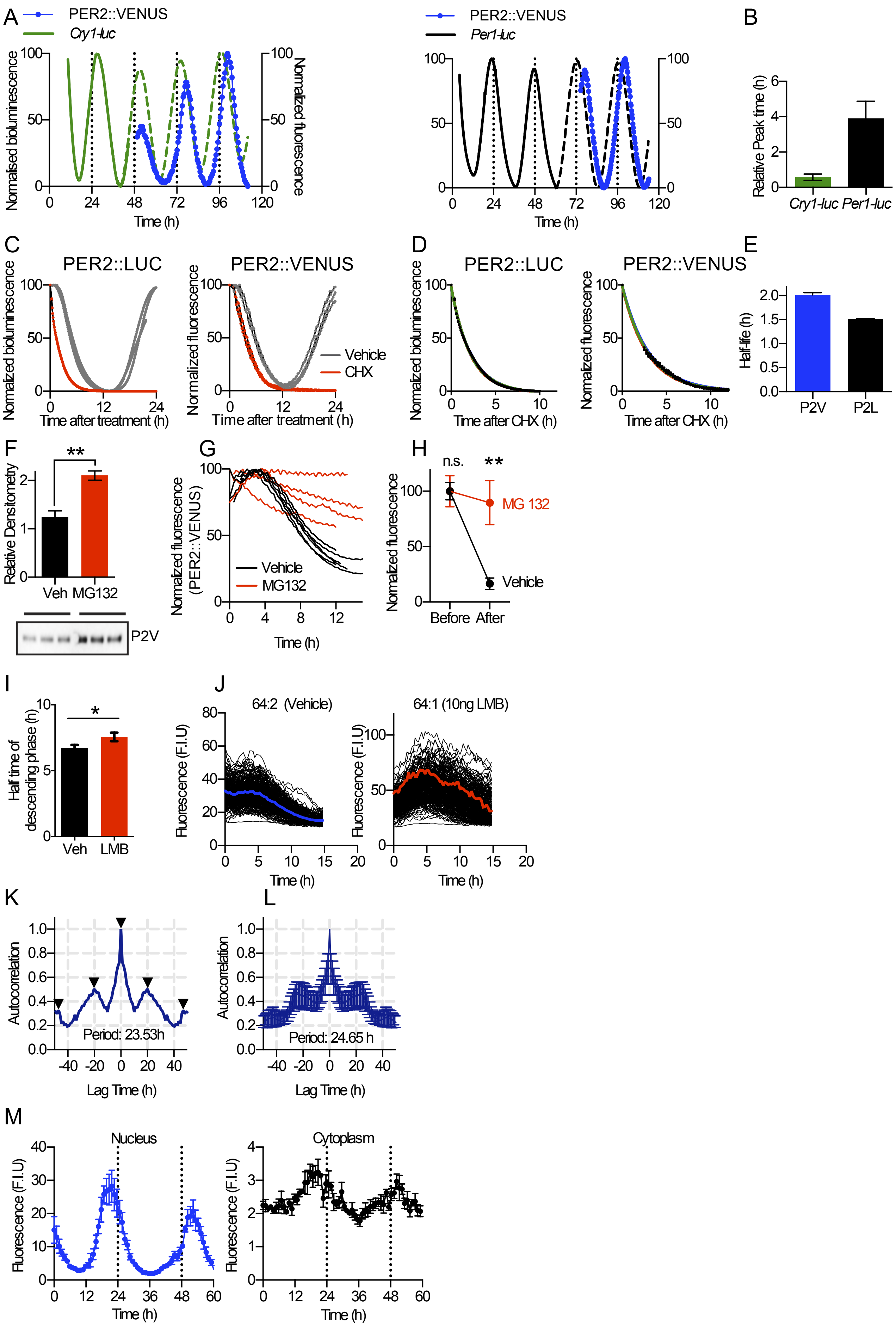

A
PER2::VENUS
Cry1-luc
Normalised bioluminescence
Normalized fluorescence
Time (h)
PER2::VENUS
Per1-luc
Time (h)
B
Relative Peak time (h)
Cry1-luc
Per1-luc
C
PER2::LUC
PER2::VENUS
Normalized bioluminescence
Normalized fluorescence
Time after treatment (h)
Vehicle
CHX
D
PER2::LUC
PER2::VENUS
Time after CHX (h)
E
Half-life (h)
P2V
P2L
F
Relative Densitometry
**
Veh
MG132
P2V
G
Normalized fluorescence (PER2::VENUS)
Vehicle
MG132
Time (h)
H
n.s.
**
MG 132
Vehicle
Normalized fluorescence
Before
After
I
Half time of descending phase (h)
*
Veh
LMB
J
64:2 (Vehicle)
64:1 (10ng LMB)
Fluorescence (F.I.U)
Time (h)
K
L
Autocorrelation
Period: 23.53h
Period: 24.65 h
Lag Time (h)
M
Nucleus
Cytoplasm
Fluorescence (F.I.U)
Time (h)

Fig. S3. Probing PER2::VENUS macrodynamics using reporters and pharmacological agents. Related to Figure 3.

(A) Representative traces showing consecutive recording of *Cry1-luc* (left) or *Per1-luc* (right) reporter bioluminescence measured by PMT followed by PER2::VENUS fluorescence (blue) captured by confocal microscopy, within the same slice. Dotted lines represent extrapolated rhythms of the bioluminescence reporters. (B) Phase-mapping circadian reporters on to PER2::VENUS. Peak times (mean ± SEM) of reporter circadian rhythms relative to PER2::VENUS, quoted as time prior to PER2::VENUS peak. (C) 24 hour recordings of PER2::LUC bioluminescence and PER2::VENUS fluorescence in SCN slices (n = 3 per group) treated with cycloheximide (40 µM; red trace) or vehicle (grey) at the peak of Per2 expression. (D) Using the data shown in (C), a one-phase exponential decay was fitted to the "peak-to-trough" of traces (ca. first 10 h). (E) Half-life of PER2::LUC and PER2::VENUS (mean ± SEM). (F) Western blot and band relative density measure for PER2::VENUS fibroblasts treated with either vehicle (Veh) or 25 µM MG132. MG132 significantly increased abundance of PER2::VENUS (n_{veh} =3; n_{MG132} =3; Student's T-test, ** $p < 0.005$). (G) Mean PER2::VENUS fluorescence of individual SCN slices, treated with 50 µM MG132 (red) or vehicle (0.01% DMSO), at the peak of PER2::VENUS expression and tracked for 15 hours following treatment. (H) Fluorescence (mean ± SEM) at the peak (before treatment) and the trough (after 15 hours treatment of MG132 and vehicle treated SCN slices). A significant increase of fluorescence was observed in MG132 treated slices compared with vehicle treated (n =5, repeated-measures two-way ANOVA with Sidak's multiple comparisons test, ** $p < 0.005$). (I) Half-life (mean ± SEM) of descending phase of the cycle (peak to trough) was significantly increased by Leptomycin B (LMB) treatment (n_{veh} =5; n_{LMB} =4, Student's T-test, $p < 0.05$). (J) Single cell analysis of PER2::VENUS fluorescence in representative SCN slices treated with either vehicle or 10 LMB (ng/µL). Mean fluorescence is shown by colored lines. (K) Representative trace showing autocorrelation analysis applied to a nuclear fluorescence trace measured for 80 h, to calculate circadian period. Autocorrelation peak locations are marked with arrows. (L) Mean ± SD of autocorrelation data (n = 19). (M) Fluorescence intensity (mean ± SEM) measured in the nucleus (left panel) or cytoplasm (right panel) of individually tracked cells (n =3) across 60 hours of recording.

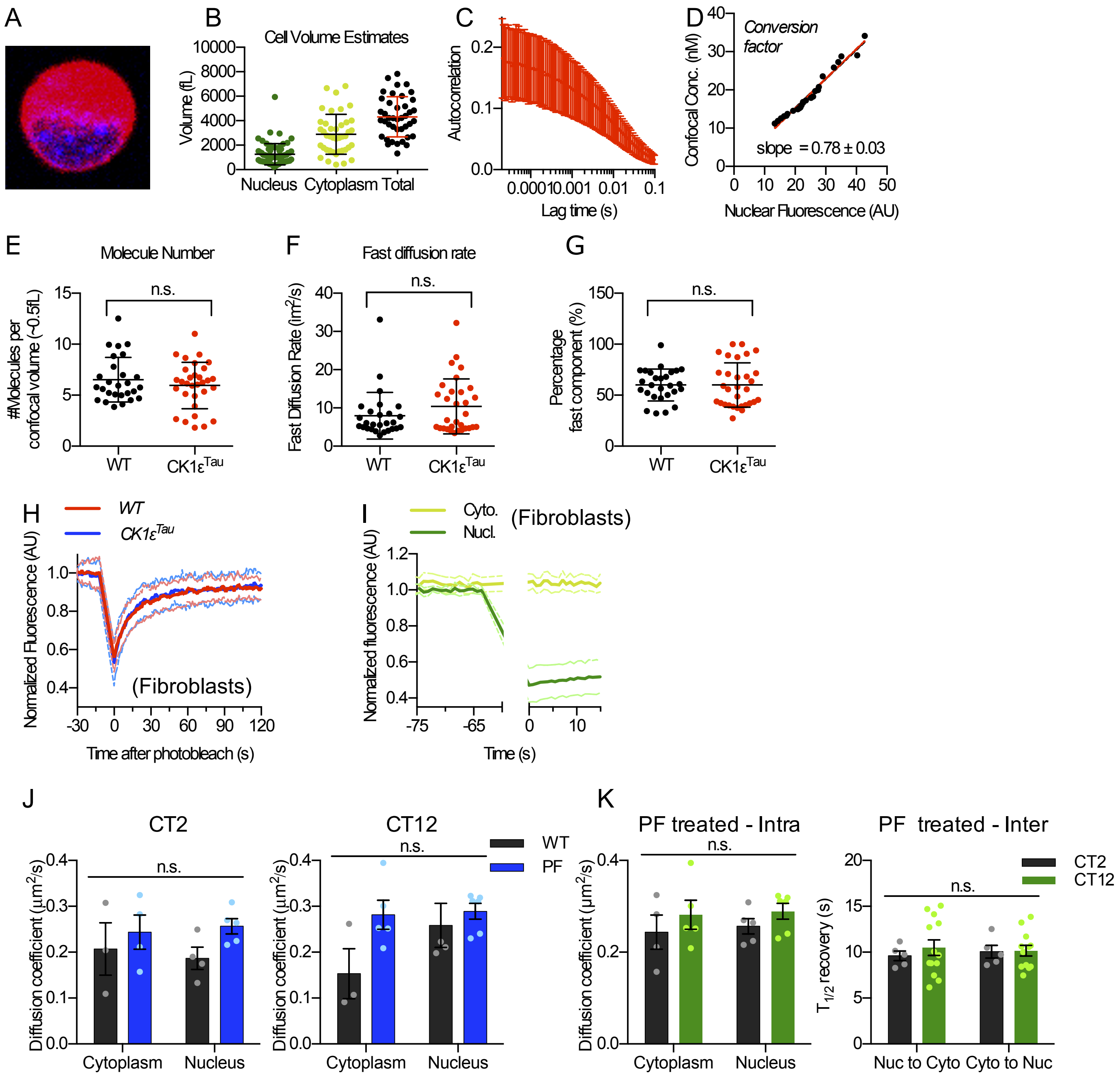
A
B
Cell Volume Estimates
Volume (fL)
Nucleus Cytoplasm Total
C
Autocorrelation
Lag time (s)
D
Conversion factor
Confocal Conc. (nM)
slope = 0.78 ± 0.03
Nuclear Fluorescence (AU)
E
Molecule Number
#Molecules per confocal volume (~0.5fL)
n.s.
WT
CK1ε^Tau
F
Fast diffusion rate
Fast Diffusion Rate (im²/s)
n.s.
WT
CK1ε^Tau
G
Percentage fast component (%)
n.s.
WT
CK1ε^Tau
H
WT
CK1ε^Tau
Normalized Fluorescence (AU)
(Fibroblasts)
Time after photobleach (s)
I
Cyto.
Nucl.
(Fibroblasts)
Normalized fluorescence (AU)
Time (s)
J
CT2
CT12
WT
PF
n.s.
Diffusion coefficient (μm²/s)
Cytoplasm
Nucleus
K
PF treated - Intra
PF treated - Inter
CT2
CT12
n.s.
Diffusion coefficient (μm²/s)
T1/2 recovery (s)
Cytoplasm
Nucleus
Nuc to Cyto
Cyto to Nuc

Figure S4. Quantitative measures of PER2::VENUS intracellular dynamics. Related to Figures 3 and 4.

(A) Representative z-stack image of a dissociated (using trypsin) skin fibroblast. Cytoplasm is stained with CellTracker™ Red CMTPX and cell nucleus with Hoechst 33342, used to calculate cell volume. (B) Nuclear and cytoplasmic volume measurements (n =41 cells). (C) Autocorrelation curve for the peak of PER2::VENUS expression (mean ± SD, n =27). (D) Quartile-quartile plot comparison between peak confocal concentrations and mean peak nuclear fluorescence, used as a calibration for converting fluorescence intensity into PER2::VENUS concentration. (E) Number of molecules in confocal volume. The confocal volume had previously been estimated at 0.59 ± 11 fL using Rhodamine 6G. The *CK1ε* ^{Tau} allele had no effect on the numbers of PER2::VENUS molecules in the nucleus (n_{WT} =28; n_{Tau} =31; T-test). (F) "Fast" component (as defined in Fig.3E) diffusion rate was not significantly different between *WT* and *Tau* mutant fibroblasts (n_{WT} =19; n_{Tau} =25; T-test). (G) Proportion of cells displaying "fast" component is not significantly different between WT and Tau mutant fibroblasts (n_{WT} =27; n_{Tau} =31 cells; T-test). All above group data are mean ± SEM. (H) Normalized fluorescence intensity (mean ± SD) in bleached nuclear ROI (5µm diameter circle) for the recovery of photobleached WT (red, n = 19) and *CK1ε*^{Tau} (blue, n =25) temperature synchronized fibroblasts. (I) Fluorescence recovery curves (mean ± SD, blue line: n =14, red line: n =13) observed following nuclear ROI fluorescence bleaching (5µm diameter). (J) PF670462 treatment (1 uM) does not alter PER2::VENUS mobility within (intra-compartment) the cytoplasm or nucleus at CT2 (left) or CT12 (right), compared to WT (CT2: $n_{WT,cyto}$ =3; $n_{PF,cyto}$ =5; $n_{WT,nuc}$ =4; $n_{,PF,nuc}$ =6; CT12: $n_{WT,cyto}$ =4; $n_{PF,cyto}$ =5; $n_{WT,nuc}$ =5; $n_{,PF,nuc}$ =6; two-way ANOVA). (K) Intra-compartment (left) and inter-compartment (right) mobility of PER2::VENUS during PF670462 treatment. Note that same intra-compartment data are shown in separate graphs in (J) (blue bars). Intra-compartment mobility of PER2::VENUS does not change between CT, or compartment during treatment ($n_{CT2,cyto}$ =4; $n_{CT12,cyto}$ =5; $n_{CT2,nuc}$ =5; $n_{CT12,nuc}$ =6; two-way ANOVA). Inter-compartment mobility of PER2::VENUS does not change between CT, nor direction of movement Nucleus-to-cytoplasm or cytoplasm-to-nucleus) during treatment ($n_{CT2,nuc->cyto}$ =5; $n_{CT12,nuc->cyto}$ =13; $n_{CT2,cyto->nuc}$ =5; $n_{CT12,cyto->nuc}$ =12; two-way ANOVA).

Table S1. Percentage of neuropeptidergic cell type that express PER2::VENUS at different times of day. Related to Figure 1. (*n* = total number of brain sections, n_{brains} = 4)

	VIP			AVP			GRP		
ZT	*n*	Mean	SEM	*n*	Mean	SEM	*n*	Mean	SEM
0	10	0	0	10	3.0	1.4	10	0	0
12	7	99.2	0.46	10	99.3	0.58	8	7.1	3.5

Supplemental experimental procedures

Animals

All animals were cared for in accordance with the UK Animals (Scientific Procedures) Act of 1986 with local ethical approval. *CK1ε*^{Tau} mice were used as previously described [S1]. *Per1-luc* reporter mice were acquired from Hitoshi Okamura (Kyoto University, Japan) [S2]. *Per1*$^{-/-}$ mice were obtained from David Weaver (UMASS Medical School, Worcester, MA, USA) [S3].

Generation of PER2::VENUS animals

A *Venus-Neomycin* construct (Figure S1) was assembled and sent to Biocenter Oulu (University of Oulu, Finland) for generation of knock-in mice using a gene-targeting strategy in 129Sv embryonic stem (ES) cells. Resulting ES cells were injected into blastocyst stage C57Bl6 embryos. The F1 generation mice were tested for the presence of *Per2*$^{Venus\text{-}Neo}$ by Southern blot. *Neo* was removed by crossing with a cre-recombinase deleter mouse line. Mice from the F1 generation of this cross were genotyped for the presence of *Per2*^{Venus} and the loss of *Neo.* Positive mice were then crossed with C57/BL6 mice (>6 generations) and subsequently bred to give homozygosity for the *Per2*^{Venus} allele.

Primers:

P1: CTGTGTTTACTGCGAGAGT (*Per2* exon 23)

R1: GGGTCCATGTGATTAGAAAC (*Per2*^{WT})

R2: TTGAAGAAGTCGTGCTGC (*Per2*^{Venus})

Wheel-running

Mice were entrained to a 12:12 light-dark (LD - 45-50 lux with white light and 5 lux in red light) cycle for at least 10 days prior to a schedule of continuous dim red light (DD) for 14 days. Data were analyzed using ClockLab (ActiMetrix Inc., USA) running within Matlab (Mathworks, USA), as previously described [S4]. As well as circadian period, we calculated the daily activity patterns (from 3 days of DD activity data), which were then normalized, aligned and plotted as mean profiles for *WT* and *Per2^Venus^* genotypes. Non-Parametric circadian rhythm analysis (NPCRA) was applied to 12 days ("days" were adjusted to match the circadian period of the individual animal) of wheel-running data, which generated the following parameters:

1. **Relative Amplitude (RA)** – Non-Parametric measure of rhythm amplitude between the mean lowest activity and mean highest daily activity measured over 5-hour and 10-hour time-windows, respectively.

$$RA = (Max_{average} - Low_{average}) / (Max_{average} + Low_{average})$$

2. **Intra-daily Variability (IV)** – Non-Parametric measure of frequency of activity/rest transitions. Can range between 0 (data fits Sine wave) to 2 (Gaussian noise).

3. **Inter-daily Stability (IS)** – how well daily activity patterns match on day-to-day basis. Can range from 0 (Gaussian noise) to 1 (high-stability).

These collectively give an indication of circadian coherence and robustness. Robust, rhythmic behavior would be characterized as having a high RA, low IV and high IS.

Fibroblast culture and temperature entrainment

Primary cultured lung fibroblasts from PER2::VENUS mice were seeded in DMEM/10 % FBS/Penstrep and split in a 1:2 ratio until passage 6 and then seeded into 3.5 cm dishes at a confluence ~ 300000 cells/dish. Once cells had attached, medium was changed and dishes transferred to an 5% CO_2 incubator set to alternate with 12h cycles of high (37 °C) and low (32 °C) temperature for four cycles. The samples were then transferred to constant high temperature (37 °C). For imaging, cells were processed 4-6 h after transfer to 37 °C and for Western blots, cells were harvested every four hours for 48 h. Dishes were washed in cold PBS before being frozen down at -80 °C and stored briefly before lysis and further processing. Each dish was lysed in 50 µL cold lysis buffer: 1 % NP40/ 0.25% sodium deoxycholate/ 50 mM Tris/ 1x Roche Protease Inhibitor Cocktail. The lysates were sonicated on ice for 30 seconds and a soluble protein fraction was obtained by centrifuging for 10 min at 21000 *g*. The protein lysates were normalized by BCA protein assay (Pierce, USA).

Gel electrophoresis and Western blotting

Protein lysates were subject to gel electrophoresis using NuPAGE Novex 4–12% Bis-Tris gradient gels (Life Technologies, USA), and run as per the manufacturer’s protocol, using the MOPS buffer system. A wet tank system was used for protein transfer to PVDF membrane (30 V for 1 h at 4 °C). PVDF membranes were blocked for 1 hour (0.5% w/w non-fat dried milk (Marvel, USA) in TBST). Membranes were then incubated with anti-rabbit GFP antiserum (1:10000, Abcam, U.K.) overnight at 4 °C, followed by three 20 min washes. The ECL Prime (GE Healthcare, USA) chemiluminescence detection system and imaged using the GelDoc system (Biorad, USA). To check protein loading was even in the gels, duplicate gels were stained with

Coomassie InstantBlue (Expedeon, uSA). Densiometric quantification of Western blots was performed by Image Lab (Bio Rad, USA).

Immunofluorescence

PFA-fixed (4% PFA in phosphate buffer) SCN sections (40 μm) were blocked in 5% normal serum, matched to the primary antisera, in blocking buffer (BSA 1%, 0.3% Triton X-100 in PBS) for one hour, with gentle shaking. Sections were incubated with primary antisera overnight, at 4 °C, with gentle shaking. They were then washed twice for 5 minutes and incubated with secondary antiserum (Alexa 647, 1:500, Life Technologies, USA) for one hour at room temperature. Sections were then washed twice for 5 minutes in wash buffer (BSA 1%, 0.3% Triton X-100 in PBS) and then twice for 5 minutes in PBS. Sections were mounted with Vectashield with DAPI (Vectorlabs, USA), ready for confocal imaging. Primary antisera used: rabbit anti-AVP (1:1000; Bachem, USA), guinea-pig anti-VIP (1:1000, Bachem, USA) and rabbit anti-GRP (1:1000; Immunostar, USA). Both immunofluorescence and native VENUS florescence were imaged using a Zeiss 780 inverted confocal set-up (Zeiss, Germany). Low magnification images were acquired using a 20x objective and high magnification images were acquired using a 63x oil objective.

Fluorescence co-localization analysis

Semi-automated cell counts were made using the “Nucleus Counter” ImageJ (NIH, USA) plugin [S5]. Mander’s co-localisation analysis was conducted using the Mander’s Coefficient plugin within ImageJ. This analysis generates a table containing the Mander’s coefficient as well as values for M1 and M2, which distinguish the co-localisation of channel 1 with channel 2 and channel 2 with 1. For example, all of

channel 1 may co-localize with channel 2, but only 50% of channel 2 may co-localize with channel1, in which case M1 =1.0, M2 =0.5.

Organotypic slices

SCN slice cultures were made as previously described [S6] and kept in culture for 3 days prior to real-time confocal imaging, or 7 days prior to bioluminescence recordings. Whole-slice bioluminescence emissions were detected by photon multiplier tubes (Hamamatsu, Japan), set-up to count emitted photons every second in 6 minute bins.

Real-time confocal microscopy

Real-time imaging of fibroblasts and SCN slices was conducted using the Zeiss LSM780 inverted system (Zeiss, Germany), with a heated chamber kept at 37 °C. Samples were placed in air-tight, glass-bottom imaging dishes (Mattek, USA). For long-term imaging, a 10x objective was used, and images acquired 2 frames per hour (fph), 30 seconds scan time per frame, for ~70 hours. Fibroblasts were imaged for ~ 70 hours at 6 fph, with a pixel dwell time of 1.58 μs.

Drug treatments

Temperature-entrained primary lung fibroblast at peak PER2::VENUS expression (~4 h after 37˚C→32˚C) were incubated for 6 h in either 25 μM MG132 proteasome inhibitor (Cayman Chemical Company, USA) or vehicle. Lysis etc. was performed as described as above. Prior to all pharmacological treatments of $Per2^{Venus}$ SCN slices, "pre-treatment" fluorescence measures were acquired by confocal microscopy. Confocal image time-series were then acquired once drug treatments were applied to the slices, imaged at a rate of 4 frames per hour. In SCN slices, cycloheximide (Sigma Aldrich, USA) was used at 40 μM,

leptomycin B (Sigma Aldrich, USA) at 10 ng/mL and MG132 at 50 μM. Imaging data were either analyzed using mean fluorescence of the whole SCN or single cell fluorescence from the slice. The latter employed the SARFIA package [S7] running within Igor Pro (Wavemetrics, USA) to select "cell-like" regions of interest.

Fluorescence Correlation Spectroscopy and Quantified Time-lapse Imaging

FCS and quantified time-lapse were conducted as previously described [S8], using a Zeiss LSM780 on an Axio observer Z1 microscope using a plan-apochromat 63x NA 1.4. VENUS fluorescence was excited by 514 nm laser light and emission collected between 518 and 535nm after passing through a pinhole set to one Airy unit. Laser power was typically <5% total power, but was adjusted as necessary to avoid photo-bleaching and also to give a suitable count rate with a minimum 1kHz counts per molecule. The protocols as outlined in Kim et al. [S9] were followed with 10x10s runs used for each measurement. Zen 2010B software was used for data collection. A custom made script for data fitting was written in MATLAB R2014b using the Optimisation Toolbox based on the Marquardt-Levenberg algorithm. The distribution of FCS-measured peak concentration was also used to calibrate confocal data sets by quantile–quantile matching to a respective distribution of peak fluorescence as determined by image analysis.

Fluorescence recovery After Photobleaching (FRAP)

SCN slices were cut from their membrane insert on which they had been cultured, and positioned facedown on to the imaging glass to bring the tissue within the working distance of the 40x objective.

FRAP was conducted with a Zeiss LSM780, using a 40x water immersion objective. SCN slices used for FRAP experiments were either with or without casein kinase 1 inhibitor (PF670462) at 1 μM. FRAP measurements were taken at either CT2 or CT12. Four different types of photobleach were used: whole nucleus, 3 μm diameter disc in the nucleus (part nucleus), whole cytoplasm, 3 μm diameter disc in the cytoplasm (part cytoplasm). For each, the following protocol was used: 10 consecutive pre-bleach images acquired at low laser intensity (10%), followed by 100 times repeat exposure to photo-bleach at 100% laser intensity, and post-bleach image acquisition at 10 frames per second.

FRAP data analysis

FRAP data were processed to remove background noise, corrected for acquisition bleaching and normalized to 100%. The $t_{1/2}$ of recovery was calculated and diffusion coefficient estimated using a simple diffusion model [S10] given by: $D = 0.88w^2 / 4\ t_{1/2}$.

These steps are outlined below:

Mean fluorescence intensity was measured from the selected bleach area (Y_{raw}), outside the cell (Y_{bg}) and different unbleached cell (Y).

1. Background subtraction: The raw data from the bleach spot was first background subtracted (Y_{bs}) using the background measurement:

$$Y_{bs} = Y_{raw} - Y_{bg}$$

2. Calculate acquisition bleaching correction factor: Y_{pc} was fitted to a one-phase decay non-linear regression given by:

$$Y = Y_0(exp(-kt)$$

where Y is Y_{pc}, Y_0 is Y_{pc} at $t = 0$, k is the acquisition correction factor and t is time in seconds.

3. Acquisition bleaching correction: The calculated value for k was used in the following equation and applied to background subtracted data:

$$Y_{pc} = Y_{bg} + (Y_{bg,t=0} - (Y_{bg,t=0} (exp(-kt))))$$

Where Y_{pc} is photo-bleach corrected signal, Y_{bg} is background subtracted signal and $Y_{bg,t=0}$ is background subtracted signal at $t = 0$

4. Normalization: The value for Y_{pc} is normalized so that Y_{pc} at time = 0 is 100%

5. Normalized data was fit to the standard FRAP equation:

$$Y = A(1- exp(-k_2t)$$

where the value for k_2 used calculate the $t_{1/2}$ of fluorescence recovery:

$$D = 0.88w^2 / 4t_{1/2}$$

Circadian analyses of bioluminescence rhythms

Bioluminescence rhythm data generated from PMTs were analyzed using non-linear fast-Fourier transform (NL-FFT) as part of circadian analysis software running within Biodare (Prof. A. Millar, University of Edinburgh; www.biodare.ed.ac.uk). To avoid potential initialization artifacts in the analyses, the first 24 hours of the recordings were omitted. Circadian period, amplitude relative phase and relative amplitude error (RAE) were calculated for each dataset.

Statistical tests

All graphs and statistics were generated using Prism (Graphpad, USA), unless specified. Datasets containing two groups were tested by Student's two-tailed t-test. Data composed of three or more groups and a treatment variable were analyzed by two-way ANOVA with Tukey's post-hoc multiple comparison test.

Supplemental References

S1. Meng, Q.J., Logunova, L., Maywood, E.S., Gallego, M., Lebiecki, J., Brown, T.M., Sladek, M., Semikhodskii, A.S., Glossop, N.R.J., Piggins, H.D., et al. (2008). Setting clock speed in mammals: The CK1 epsilon tau mutation in mice accelerates circadian pacemakers by selectively destabilizing PERIOD proteins. Neuron *58*, 78-88.

S2. Yamaguchi, S., Mitsui, S., Miyake, S., Yan, L., Onishi, H., Yagita, K., Suzuki, M., Shibata, S., Kobayashi, M., and Okamura, H. (2000). The 5 ' upstream region of mPer1 gene contains two promoters and is responsible for circadian oscillation. Curr Biol *10*, 873-876.

S3. Bae, K., Jin, X.W., Maywood, E.S., Hastings, M.H., Reppert, S.M., and Weaver, D.R. (2001). Differential functions of mPer1, mPer2, and mPer3 in the SCN circadian clock. Neuron *30*, 525-536.

S4. Maywood, E.S., Chesham, J.E., Meng, Q.J., Nolan, P.M., Loudon, A.S.I., and Hastings, M.H. (2011). Tuning the Period of the Mammalian Circadian Clock: Additive and Independent Effects of CK1 epsilon(Tau) and Fbxl3(Afh) Mutations on Mouse Circadian Behavior and Molecular Pacemaking. J Neurosci *31*, 1539-1544.

S5. Collins, T.J. (2007). ImageJ for microscopy. Biotechniques *43*, 25-30.

S6. Hastings, M.H., Reddy, A.B., McMahon, D.G., and Maywood, E.S. (2005). Analysis of circadian mechanisms in the suprachiasmatic nucleus by transgenesis and biolistic transfection. Method Enzymol *393*, 579-592.

S7. Dorostkar, M.M., Dreosti, E., Odermatt, B., and Lagnado, L. (2010). Computational processing of optical measurements of neuronal and synaptic activity in networks. J Neurosci Meth *188*, 141-150.

S8. Bagnall, J., Boddington, C., Boyd, J., Brignall, R., Rowe, W., Jones, N.A., Schmidt, L., Spiller, D.G., White, M.R.H., and Paszek, P. (2015). Quantitative dynamic imaging of immune cell signalling using lentiviral gene transfer. Integrative Biology *7*, 713-725.

S9. Kim, S.A., Heinze, K.G., and Schwille, P. (2007). Fluorescence correlation spectroscopy in living cells. Nature methods *4*, 963-973.

S10. Axelrod, D., Koppel, D.E., Schlessinger, J., Elson, E., and Webb, W.W. (1976). Mobility Measurement by Analysis of Fluorescence Photobleaching Recovery Kinetics. Biophys J *16*, 1055-1069.
